# Reproductive options for families at risk of Osteogenesis Imperfecta: a review

**DOI:** 10.1186/s13023-020-01404-w

**Published:** 2020-05-27

**Authors:** Lidiia Zhytnik, Kadri Simm, Andres Salumets, Maire Peters, Aare Märtson, Katre Maasalu

**Affiliations:** 1 Clinic of Traumatology and Orthopaedics, Tartu University Hospital, Tartu, Estonia; 2 Institute of Philosophy and Semiotics, Faculty of Arts and Humanities, University of Tartu, Tartu, Estonia; 3 Centre of Ethics, University of Tartu, Tartu, Estonia; 4 Competence Centre on Health Technologies, Tartu, Estonia; 5 Department of Obstetrics and Gynaecology, Institute of Clinical Medicine, University of Tartu, Tartu, Estonia; 6 Institute of Genomics, University of Tartu, Tartu, Estonia; 7 COMBIVET ERA Chair, Institute of Veterinary Medicine and Animal Sciences, Estonian University of Life Sciences, Tartu, Estonia; 8 Department of Traumatology and Orthopaedics, Institute of Clinical Medicine, University of Tartu, Tartu, Estonia

**Keywords:** Reproduction, Osteogenesis Imperfecta, Family planning, Bone fragility, Prenatal diagnosis, Preimplantation genetic testing, Preconception carrier screening, Ethical decision-making, Ethics of prenatal testing

## Abstract

**Background:**

Osteogenesis Imperfecta (OI) is a rare genetic disorder involving bone fragility. OI patients typically suffer from numerous fractures, skeletal deformities, shortness of stature and hearing loss. The disorder is characterised by genetic and clinical heterogeneity. Pathogenic variants in more than 20 different genes can lead to OI, and phenotypes can range from mild to lethal forms. As a genetic disorder which undoubtedly affects quality of life, OI significantly alters the reproductive confidence of families at risk. The current review describes a selection of the latest reproductive approaches which may be suitable for prospective parents faced with a risk of OI. The aim of the review is to alleviate suffering in relation to family planning around OI, by enabling prospective parents to make informed and independent decisions.

**Main body:**

The current review provides a comprehensive overview of possible reproductive options for people with OI and for unaffected carriers of OI pathogenic genetic variants. The review considers reproductive options across all phases of family planning, including pre-pregnancy, fertilisation, pregnancy, and post-pregnancy. Special attention is given to the more modern techniques of assisted reproduction, such as preconception carrier screening, preimplantation genetic testing for monogenic diseases and non-invasive prenatal testing. The review outlines the methodologies of the different reproductive approaches available to OI families and highlights their advantages and disadvantages. These are presented as a decision tree, which takes into account the autosomal dominant and autosomal recessive nature of the OI variants, and the OI-related risks of people without OI.

The complex process of decision-making around OI reproductive options is also discussed from an ethical perspective.

**Conclusion:**

The rapid development of molecular techniques has led to the availability of a wide variety of reproductive options for prospective parents faced with a risk of OI. However, such options may raise ethical concerns in terms of methodologies, choice management and good clinical practice in reproductive care, which are yet to be fully addressed.

## Background

Procreation is one of the declared meanings of life [1]. Numerous studies have shown a positive association between the ability to have offspring and satisfaction with quality of life (QoL) in both men and women and across different cultures [2]. Since reproduction comprises such an important aspect of a person’s life, it can raise challenging issues for those who suffer from infertility or a genetic disorder. Having a genetic disorder strongly affects the reproductive decisions of an affected individual or a carrier [3]. The more severe, the earlier the onset and the less curable a genetic condition is, the more complicated and limited the reproductive options may seem. A lack of information about developments in reproductive medicine among patient and professional communities may result in a sense of fear and insecurity, and lead to non-independent reproductive decisions.

The latest advances in family planning and assisted reproduction have already led to considerable benefits for people with risk of a genetic disorder such as OI [4–7]. OI is a rare monogenic disorder of bone fragility, also known as a “brittle bone disease” [8]. The condition is characterised by easily occurring bone fractures, skeletal deformities, shortness of stature and bluish eye sclera. Patients also suffer from hearing loss, joint hypermobility, dentinogenesis imperfecta, and cardiovascular and pulmonary complications [9–13]. Due to high phenotypic variability, symptoms may vary between OI patients. Phenotypes may differ even between OI individuals with the same pathogenic variant, and between affected members of a single family [14, 15]. Despite being a rare genetic disorder, OI does not influence the fertility of affected individuals [16].

In most populations, one individual per 10–20,000 is affected, making OI one of the most common rare skeletal dysplasias [9, 10]. To date, no cure for OI is available: management includes the medical and surgical treatment of both skeletal abnormalities and other symptomatic complications [17–20]. The most popular OI pharmacological treatment is an anti-resorptive therapy with bisphosphonates, however this is of limited effectiveness [21]. Other options include the use of anabolic therapies (growth hormones) which, similarly to anti-resorptive approaches, support the increase of bone mass; however they do not improve bone quality [20]. A number of other therapies are currently under development, including monoclonal antibodies, gene therapies, mesenchymal stem cell transplantation and 4-phenylbutyric acid therapy [22–24]. Given the limited efficiency of existing treatments, the presence of OI can significantly affect QoL, and lead to emotional, social, physical, reproductive and health-related challenges [25–27]. Life expectancy depends on the severity of the condition, which can range from mild osteopenia to severe, perinatally lethal forms [9]. In general, patients with OI have a higher lifelong mortality rate as compared to the general population, due to respiratory, gastrointestinal and cardiovascular diseases and traumas [28].

**Fig. 1:**
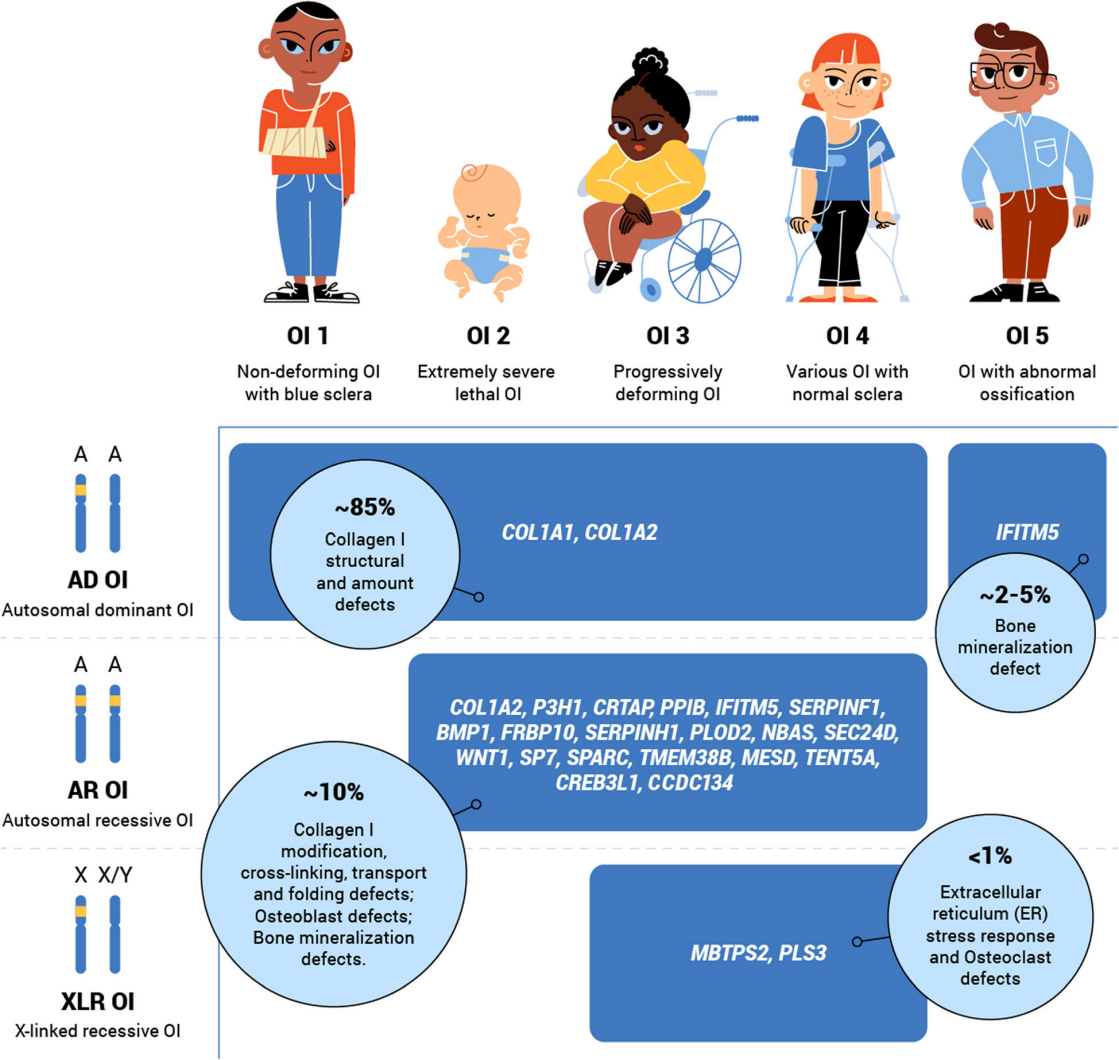
OI clinical and genetic heterogeneity. OI clinical variability ranges from mild non-deforming OI to severe and lethal OI forms. Genetic diversity of the disorder is characterised by OI pathogenic variants in more than 22 different genes. Autosomal dominant (AD), autosomal recessive (AR) and X-linked recessive (XLR) inheritance patterns were observed among OI families. A – Autosomal chromosome; OI – Osteogenesis Imperfecta; X – X chromosome; Y – Y chromosome

Clinical OI classification distinguishes between five OI types (OI 1–5) [9, 29]. OI 1 (Online Mendelian Inheritance in Man (OMIM) 166,200) is a non-deforming mild OI with blue sclera. OI 2 (OMIM 166210) is the most severe, perinatal lethal form of OI. OI 3 (OMIM 259420) is a severe, progressively deforming OI. OI 4 (OMIM 166220) is a common variable OI with normal sclera [30]. OI 5 (OMIM 610967) is an OI with progressive calcification abnormalities [31] (Fig. 1). Of the five OI types, OI 1 is estimated to affect the highest proportion of the OI population (46–71%). OI 4 and 3 are each estimated to affect between 12 to 28% of the OI population [11, 28, 32]. OI 5 is estimated to affect approximately 2–5% of the OI population [33, 34] and lethal OI 2 is estimated to affect less than 12% [28, 35] (Table 1).

**Table 1:** OI clinical nomenclature and estimated prevalence of OI types

OI clinical type	Online Mendelian Inheritance in Man (OMIM)	Description of severity	Estimated prevalence [11, 28, 32–35]
OI 1	166,200	Non-deforming mild OI with blue sclera	~ 46–71%
OI 2	166,210	Perinatal lethal form of OI	~ 12%
OI 3	259,420	Severe, progressively deforming OI	~ 12–28%
OI 4	166,220	Common variable OI with normal sclera	~ 12–28%
OI 5	610,967	OI with progressive calcification abnormalities	~ 2–5%

In ~85% of cases, the disorder is caused by pathogenic variants in the *COL1A1* (OMIM 120150) and *COL1A2* (OMIM 120160) genes [11, 34, 36–38]. The *COL1A1* and *COL1A2* genes code for α1 and α2 chains of a collagen type I protein, which comprises up to 90% of the organic component of the bone and is responsible for its elastic properties. Structural and quantitative aberrations in collagen I may therefore cause bone fragility and result in fractures [39–41]. Over the last decade, 21 other OI-related genes have been discovered (genetic OI types I-XX) [18, 29, 42] (Table 2, Fig. 1).

**Table 2:** OI genetic nomenclature combined with causative genes and phenotypes

OI clinical type	Mutated gene	Genetic OI type	OMIM	Inheritance	Protein product	Phenotype
OI 1	*COL1A1*	I-IV	166,200	AD, AR	Collagen α1(I)	Non deforming OI with blue sclera; Common variable OI with normal sclera; Progressively deforming
OI 2			166,210			
OI 3			259,420			
OI 4			166,220			
OI 1	*COL1A2*	I-IV	166,200	AD, AR	Collagen α2(I)	Non deforming OI with blue sclera; Common variable OI with normal sclera; Progressively deforming
OI 2			166,210			
OI 3			259,420			
OI 4			166,220			
OI 5	*IFITM5*	V	610,967	AD	Bone-restricted interferon-induced transmembrane protein-like protein (BRIL; also known as IFITM5)	OI with calcification in interosseous membranes, hyperplastic callus, radial head dislocation or severe bone deformity with grey sclera
OI 3						
OI 3	*SERPINF1*	VI	613,982	AR	Pigment epithelium-derived factor (PEDF)	Progressively moderate to severe deforming, osteoid, fish-scale appearance of bone lamella
OI 3	*CRTAP*	VII	610,682	AR	Cartilage-associated protein (CRTAP)	Progressively deforming, severe rhizomelia, white sclera
OI 2						
OI 3	*P3H1 (LEPRE1)*	VIII	610,915	AR	Prolyl 3-hydroxylase 1 (P3H1)	Progressively deforming, severe rhizomelia, white sclera
OI 2						
OI 3	*PPIB*	IX	259,440	AR	Peptidyl-prolyl *cis*–*trans* isomerase B (PPIase B)	Severe bone deformity with grey sclera
OI 2						
OI 3	*SERPINH1*	X	613,848	AR	Serpin H1 (also known as HSP47)	Severe skeletal deformity, blue sclera, dentinogenesis imperfecta, skin abnormalities and inguinal hernia
OI 3	*FKBP10*	XI	610,968	AR	65 kDa FK506-binding protein (FKBP65)	Mild-to-severe skeletal deformity, normal-to-grey sclera and congenital contractures
OI 3	*BMP1*	XII	614,856	AR	Bone morphogenetic protein 1 (BMP1)	Mild-to-severe skeletal deformity and umbilical hernia
OI 3	*SP7*	XIII	613,849	AR	Transcription factor SP7 (also known as osterix)	Severe skeletal deformity with delayed tooth eruption and facial hypoplasia
OI 4						
OI 3	*TMEM38B*	XIV	615,066	AR	Trimeric intracellular cation channel type B (TRIC-B; also known as TM38B)	Severe bone deformity with normal-to-blue sclera
OI 3	*WNT1*	XV	615,220	AD, AR	Proto-oncogene Wnt-1 (WNT1)	Severe skeletal abnormalities, white sclera and possible neurological defects
OI 4						
OI 2	*CREB3L1*	XVI	616,229	AR	Old astrocyte specifically induced substance (OASIS; also known as CR3L1)	Progressively severe deforming, respiratory deficiency
OI 3						
OI 3	*SPARC*	XVII	616,507	AR	SPARC (also known as osteonectin)	Progressively severe deforming, severe fragility
OI 3	*TENT5A (FAM46A)*	XVIII	617952	AR	Terminal nucleotidyltransferase 5A	Progressively moderate to severe, congenital bowing of the lower limbs
OI 3	*MBTPS2*	XIX	301014	XLR	Membrane-bound transcription factor site-2 protease (S2P)	Progressively moderate to severe deforming, light blue sclera
OI 3	*PLOD2*	No type	609,220	AR	Lysyl hydroxylase 2 (LH2)	Progressively moderate to severe deforming, joint contractures
OI 3	*MESD*	XX	618,644	AR	Mesoderm development LRP chaperone	Progressive deforming OI, oligodontia
OI 4	*PLS3*	No type	300910	XLR	Plastin 3	Common variable OI with normal sclera, normal height
OI 3	*NBAS*	No type	614800	AR	Neuroblastoma amplified sequence	Progressively moderate to severe deforming, intellectual disability, liver failure, optic nerve atrophy
OI 3	*SEC24D*	No type	616294	AR	Protein transport protein Sec24D	Bone fragility, skull ossification defects, craniofacial dysmorphism
OI 3	*CCDC134*	No type	618,788	AR	Coiled-coil domain containing protein 134	Bone fragility, Wormian bones, limited joint mobility, pseudoarthroses

Adapted from Online Mendelian Inheritance in Man (OMIM) database [18, 29, 43]

Being a monogenic disorder with high genetic and phenotypic variability, OI raises many ethical issues around family planning, both for medical professionals and for families with a risk of OI. For example, in autosomal dominant (AD) families, OI may be passed down through many generations: due to the high probability of variant transmission (50%), there is a low chance of eliminating the disorder from the genealogical tree. Given the limited effectiveness of current treatment approaches, families with OI risk face difficulties in the realisation of their reproductive interests.

The current article provides an overview of the latest advances in reproductive options for prospective parents faced with OI, with a focus on modern reproductive medicine techniques. The article discusses reproductive approaches which may increase the likelihood of unaffected offspring in OI families. A reproductive decision tree for families at risk of OI is also presented. Finally, the article reviews some of the ethical concerns associated with OI reproductive options, in order to assist good clinical practice and to support OI families in making independent reproductive decisions.

## Main text

### Reproductive options for prospective parents faced with Osteogenesis Imperfecta

Before beginning the family planning process, prospective parents faced with a risk of OI must consider some important preliminary issues:

- Do they require offspring to be genetically related, or would a genetically non-related child also be considered?
- Is natural conception preferred, or is in vitro fertilisation (IVF) also a possibility?
- Is termination of pregnancy an option (in the case of an affected pregnancy)?
- Has the possibility of accepting a child affected with OI been considered (or only the possibility of an unaffected child)?

**Fig. 2:**
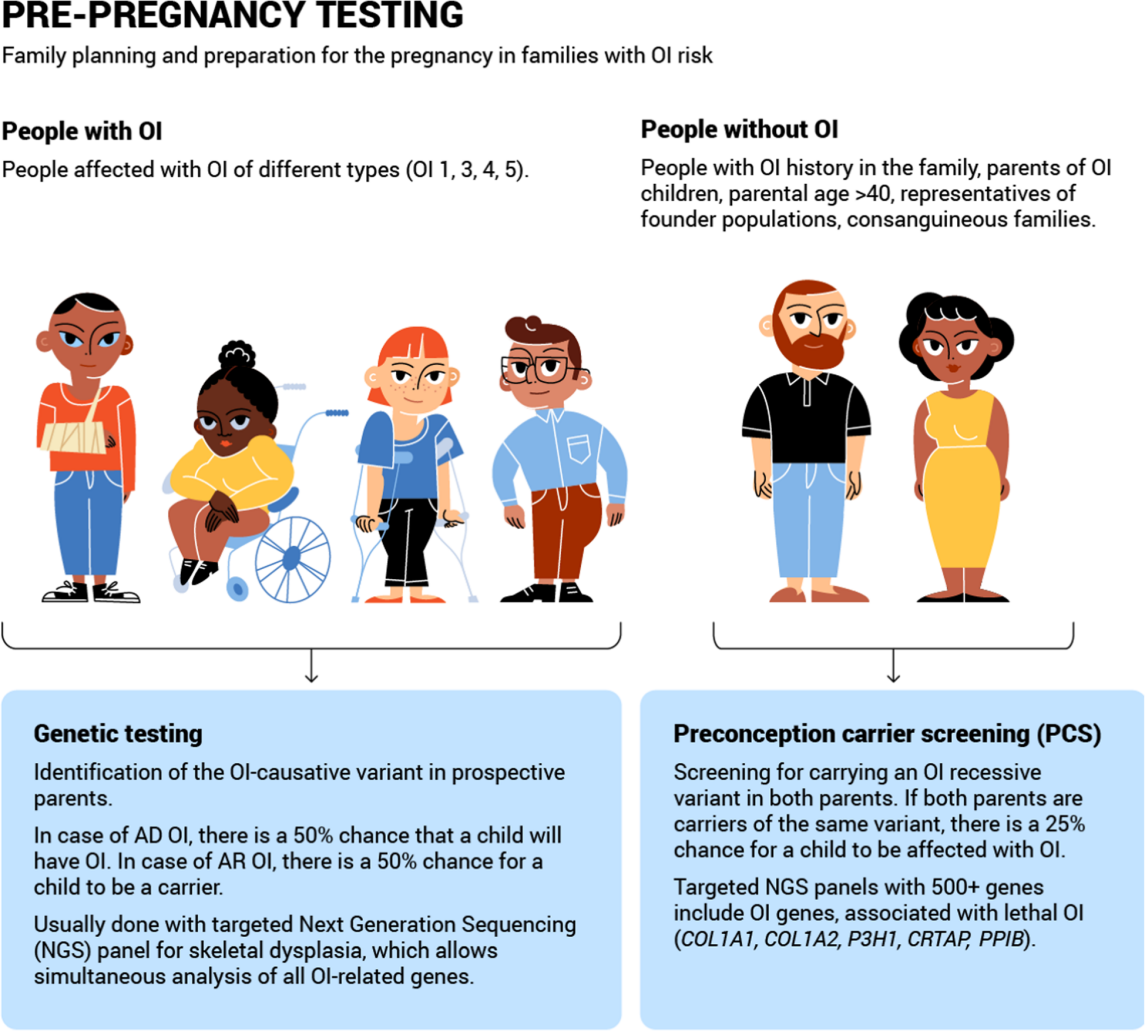
Overview of pre-pregnancy reproductive options for members of families with OI risk. Pre-pregnancy testing of OI: genetic testing and PCS - preconception carrier screening

**Fig. 3:**
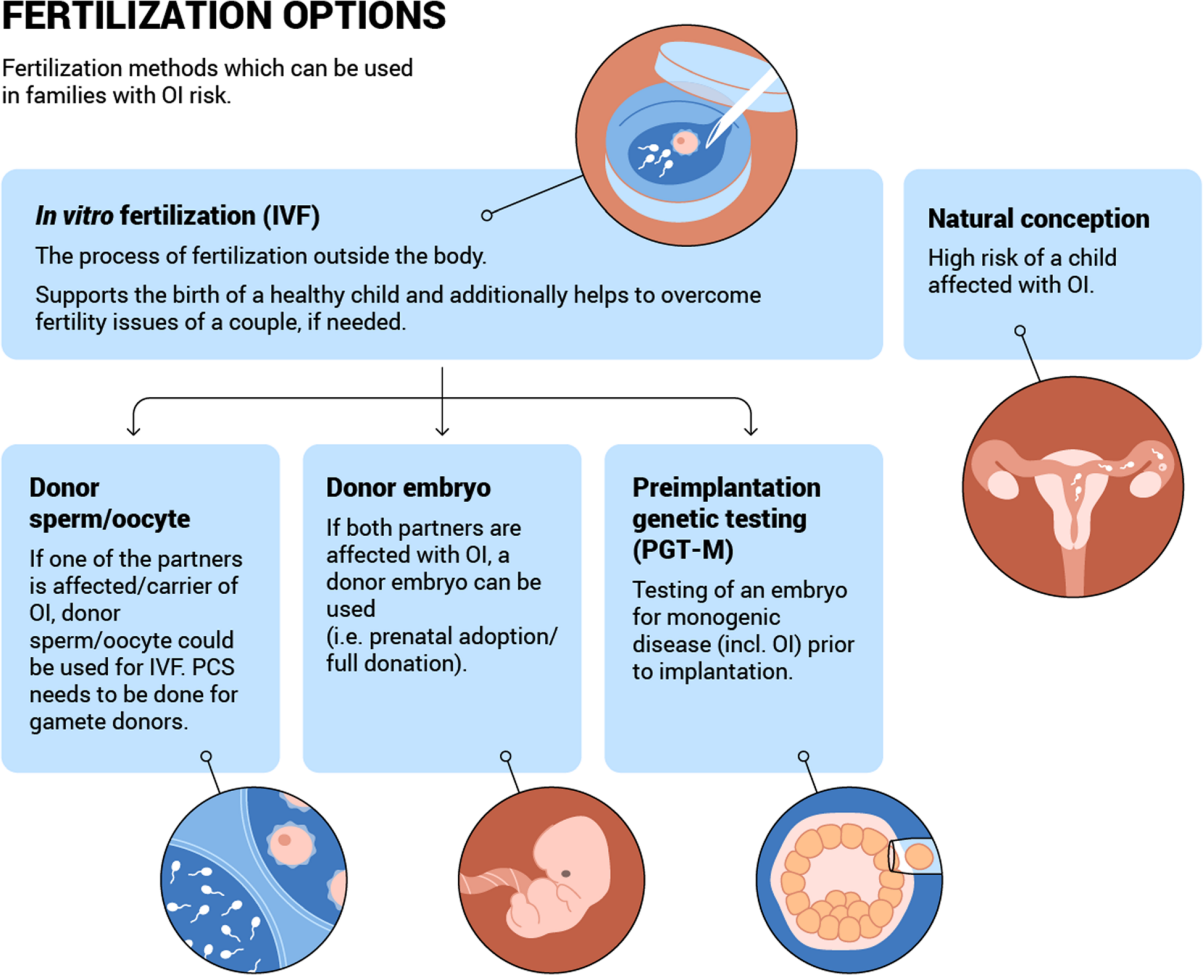
Overview of fertilisation options for couples with OI risk. IVF *-* in vitro fertilisation with donor gametes / embryo, PGT-M - preimplantation genetic testing, and natural conception

**Fig. 4:**
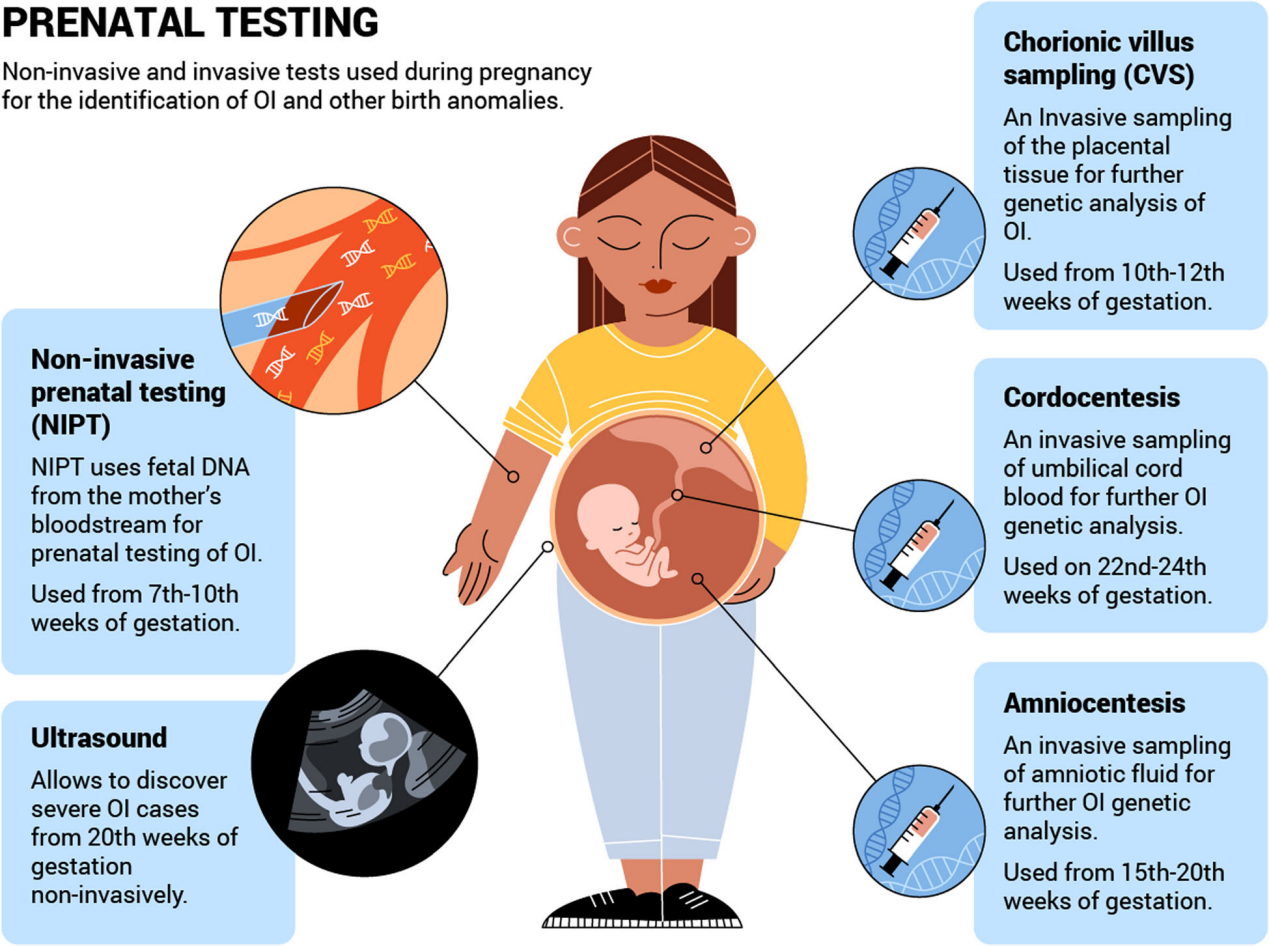
Overview of prenatal testing options for members of families with OI risk. NIPT – non-invasive prenatal testing, ultrasound, CVS - chorionic villus sampling, cordocentesis, amniocentesis

**Fig. 5:**
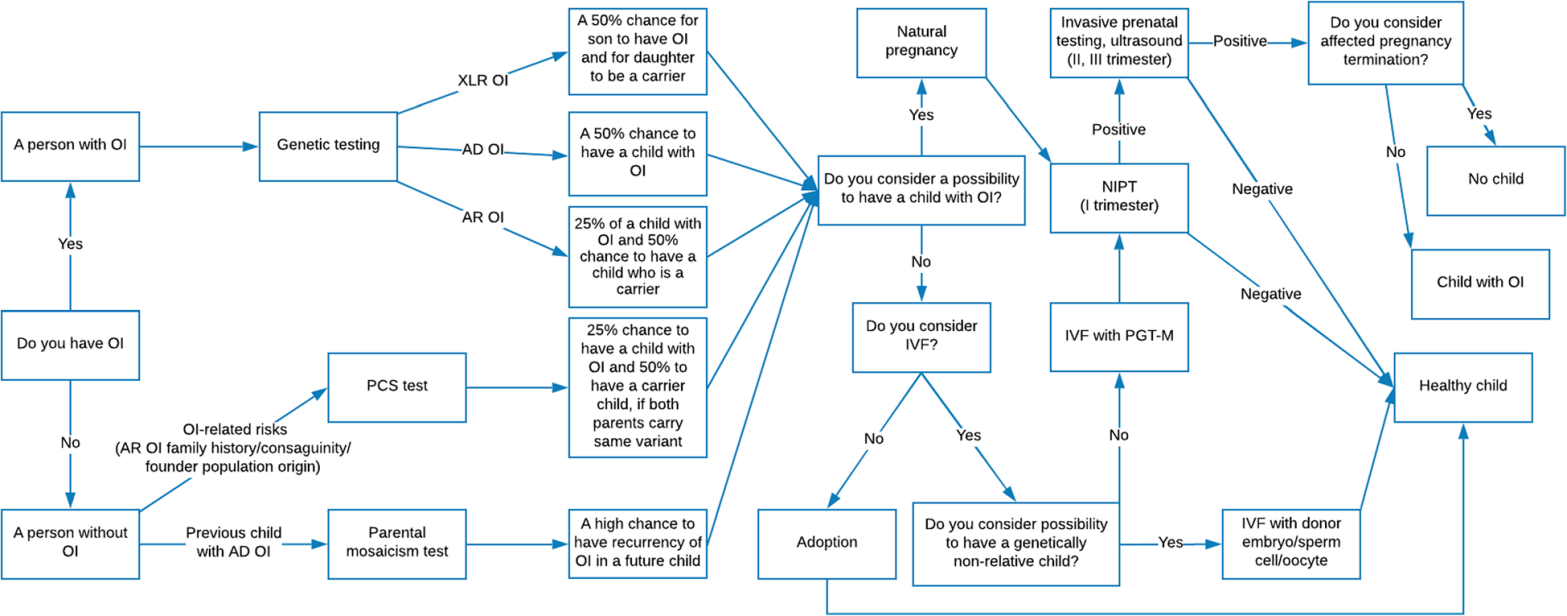
Reproductive decision tree for members of families with OI risk. Based on the OI inheritance pattern in the family, and the wishes of the prospective parents, a specific autonomous decision-supportive reproductive strategy could be chosen. AD – Autosomal Dominant; AR – Autosomal recessive; IVF – In Vitro Fertilisation; NIPT – Non-invasive Prenatal testing; OI – Osteogenesis Imperfecta; PCS – Preconception Carrier Screening; PGT-M – Preimplantation Genetic Testing for Monogenic Disease; XLR – X-linked recessive

These questions are critical to the autonomy of prospective parents’ decisions. Only after preliminary discussion with a health specialist (e.g. a general practitioner, genetic counsellor or fertility specialist) in which the views and wishes of the prospective parents are specified should a family planning strategy be chosen. Those individuals carrying, or being at risk of carrying, an OI variant and who do not wish to pass it on to their offspring have several options, each of which will be discussed in detail below (Table 3, Figs. 2, 3 and 4). Some of the methods (e.g. pre-pregnancy testing and prenatal testing) may be also useful for those couples considering natural conception. A decision tree of reproductive options for prospective parents faced with a risk of OI is presented below (Fig. 5). Although outside the scope of the current review, we note that other health risks may affect the reproductive decisions of females with OI, in particular the associated additional pregnancy risks such as cardiorespiratory complications, severe musculoskeletal pain, bone loss, uterine and placenta rupture, and blood loss during delivery [44, 45].

**Table 3:** Comparison of OI reproductive options

**Pre-pregnancy testing**					
	**Genetic Testing**			**PCS**	
Target user	People affected with OI			People with risk of OI (OI family history, consanguinity, founder population origin)	
Advantages	Fast and informative			Fast and informative	
Limitations	VUS; negative results of genetic test			Parental mosaicism; de novo OI; VUS	
**Fertilisation methods**					
	**Natural pregnancy**		**IVF with PGT-M**	**IVF with donor germ cells**	**IVF with donor embryo**
Genetically related child	Yes		Yes	No	No
Child without OI	Unknown		Yes	Yes	Yes
OI mother’s health challenges	Pregnancy, delivery		Superovulation, pregnancy, delivery	Superovulation, pregnancy, delivery	Pregnancy, delivery
Limitations	PGT-M unavailable, high chance of OI affected pregnancy, de novo variants, variable phenotypic expressivity of OI		De novo variants	De novo variants, PCS need to be done for sex cell donors	De novo variants
**Prenatal testing**					
	**NIPT**	**Ultrasound**	**CVS**	**Amniocentesis**	**Cordocentesis**
Non-invasive	Yes	Yes	No	No	No
Week of gestation	7th–10th	20th	10th–12th	15th–20th	22nd-24th
Tests foetal abnormalities, OI	Yes	Yes	Yes	Yes	Yes
Differs OI 2–3	No	No	No	No	No
Risk of misdiagnosis	Yes (placental mosaicism)	Yes (differential diagnosis)	Yes (placental mosaicism)	No	No
Limitations	Unavailable before 7th week of gestation, high mother’s BMI	Unavailable at early gestation weeks	Unavailable at early gestation weeks, risk of miscarriage ~ 1%	Unavailable at early gestation weeks, risk of miscarriage ~ 0.5–1%	Unavailable at early gestation weeks, risk of miscarriage ~ 1–2%

*BMI* Body mass index, *CVS* Chorionic villus sampling, *IVF PGT-M* In vitro fertilisation with preimplantation genetic testing for monogenic disease, *NIPT* Non-invasive prenatal testing, *PCS* Preconception carrier screening, *VUS* Variant of unknown significance

### Pre-pregnancy reproductive options for prospective parents faced with Osteogenesis Imperfecta

During the pre-pregnancy period, an individualised approach to reproductive options may be developed which incorporates not only the OI family history and OI phenotype and genotype characteristics, but also details regarding prospective parents’ reproductive health, their abilities and their wishes (Table 3, Fig. 2). Pre-pregnancy family planning is beneficial not only for those who hope to have an unaffected pregnancy, but also for those faced with the possibility of an affected pregnancy. Pre-pregnancy preparation and family planning allow for a wider variety of reproductive options, reduce associated risks and enable the arrangement of OI pregnancy, delivery and early treatment options where necessary.

#### Family planning for people with Osteogenesis Imperfecta

Disorder severity is known to alter the reproductive decisions of OI patients [46]. Approximately 56% of families with OI 1 have several generations of OI history. This may be due to undiagnosed OI cases in older generations, resulting from lack of awareness. On the other hand, conscious risking may occur where the OI phenotype is mild and has a lower impact on QoL. Half of OI 4 patients have familial OI. In contrast, only about 14% of patients with OI 3 have a previous family history of OI, which may be explained by the severe nature of this OI form [47]. At an individual patient level, the severity of the disorder is highly subjective. According to a “Voice of people with OI” statement, “A person with mild OI who has severe pain, or who has become deaf, might regard his OI as more severe than an otherwise healthy wheelchair user with OI type 3” [48]. It follows that not only those with OI 3, but also individuals with OI 4 and even OI 1 may require assistance with reproductive confidence. Consequently, all OI patients, regardless of the severity of their OI, should be offered support and a choice of reproductive strategies.

##### Osteogenesis Imperfecta genetic testing

For people with OI, genetic testing is an important starting point for family planning. Genetic testing provides insights into a patient’s OI family history and OI genotype characteristics, and allows for the connected evaluation of OI transmission risks. Modern genetic testing is performed either after clinical OI diagnosis, or in parallel. Previously, it was more common to first perform Sanger sequencing of the *COL1A1* and *COL1A2* genes [49, 50]. However, the modern conception of OI genetic diagnosis is based on the identification of OI-causative variants, using targeted next generation sequencing (NGS) of all 23 OI-related genes simultaneously (Fig. 2). Targeted NGS allows the coding sequences and flanking regions of identified genes to be screened. As a rule, OI panels include AD, autosomal recessive (AR) and X-linked (XLR) OI genes, as well as additional genes associated with other skeletal dysplasias (such as achondroplasia, Ehlers-Danlos syndrome, monogenic osteoporosis and hypophosphatasia) [51].

Although infrequent, some difficulties in OI genetic testing may arise. These include missing causative genes in the targeted NGS panel, a new unknown causative gene, exon or whole gene deletions, and intronic variants [34, 50–52]. Sometimes, current knowledge is insufficient for the identification of the pathogenicity of a found DNA change (known as variants of unknown significance (VUS)), which complicates the interpretation of results. These limitations may also cause a negative result in a genetic test [50]. In the case of a negative result for a targeted NGS-panel, other testing options are usually considered (e.g. multiplex ligation-dependent probe amplification (MLPA), whole exome sequencing (WES), biochemical analysis, whole genome sequencing (WGS) and functional studies).

##### Autosomal dominant Osteogenesis Imperfecta

Up to 90% of OI cases are caused by autosomal dominant (AD) pathogenic variants in the *COL1A1, COL1A2* genes and the *IFITM5* (OMIM 614757) gene, with a 50% probability of transmission of the pathogenic variant to the next generation [11, 34, 36, 53, 54] (Table 2, Fig. 1). All OI 5 cases are associated with a heterozygous pathogenic variant (c.-14C > T) in the 5′ untranslated region of the *IFITM5* gene, which is associated with bone mineralization [54]. However, collagen I AD cases may represent any of OI types 1–4 and display individual characteristics [29]. More than 1500 pathogenic variants in the *COL1A1* and *COL1A2* genes have been described to date [55]. Genotype-phenotype correlations remain unclear, due to the high degree of phenotypical variability, possibly associated with the influence of genetic modifiers [29, 56, 57]. In general, the more severe forms of OI are associated with structural abnormalities of the collagen type I, and characterised by Gly missense substitutions in the *COL1A1* and *COL1A2* genes [58]. The abovementioned structural aberrations of the collagen are associated with endoplasmic reticulum stress and apoptosis of osteoblasts and are therefore more deleterious [38, 59, 60]. The severity of the phenotype is also influenced by the type of substitution and the position of the variant [38]. Although the existence of “lethal clusters” of Gly substitutions was previously proposed for the *COL1A1* and *COL1A2* genes, recent reports illustrate that some of the variants located inside these clusters are associated with non-lethal OI phenotypes [37, 38, 61]. Attempts to predict the lethality of collagen I variants are challenging, as the same variants may cause both lethal and non-lethal OI cases [62, 63]. Mild OI forms are related to loss-of-function (LoF) pathogenic variants (splice site, nonsense and frameshift) of the *COL1A1* and *COL1A2* genes [38, 60, 64].

More than half (~ 60%) of AD OI variants in the *COL1A1* and *COL1A2* genes arise sporadically as de novo cases. The majority of de novo cases are missense variants, whereas inherited OI is caused mainly by LoF variants [47, 65]. As with other monogenic disorders, the incidence rate of OI de novo cases is reported to be associated with paternal age [66]. However, the phenotype severity is not influenced by the paternal (sperm cell) or maternal (egg cell) origin of the de novo variant.

According to observations by Pyott et al., in 32% of families with unaffected parents and a first child affected with perinatally lethal OI, the disorder recurred in subsequent children [67]. Recurrence of OI in subsequent offspring is explained by the AR inheritance pattern, or parental mosaicism, which is estimated to be as high as 5–8% across all OI cases [68, 69]. Mosaicism is the presence of more than one cell population with different genotypes among the gametes and / or somatic cells of an individual. The OI-causative variant is present in only a fraction of the cells of a mosaic individual. For there to be a higher OI risk in offspring, the genetic mutation causing OI must be present in a proportion of gametes. Whereas mosaic parents may reveal mild or moderate clinical symptoms of OI, or stay asymptomatic, their offspring (if developed from a germ cell with OI variant) may be fully affected, and may even develop a severe or lethal OI phenotype [67–69].

##### Autosomal recessive Osteogenesis Imperfecta

The remaining 10% of OI cases are caused by autosomal recessive (AR) pathogenic variants in collagen I and in 19 other OI genes [18, 29, 70]. AR OI genes encode proteins involved in the post-translational modification, processing, transport and cross-linking of the collagen type I. Other AR genes are involved in the bone tissue mineralization or osteoblast differentiation and functioning (Table 2, Fig. 1) [71–87]. Unlike AD OI, the distribution of AR OI differs across populations, which is thought to be due to the effects of founder populations, migration processes and cultural traditions which tolerate consanguinity [77, 88–92]. If both parents are AR OI carriers, their probability of having an affected child is 25%.

AR pathogenic variants tend to cause the development of moderate, severe, and lethal OI phenotypes. Phenotypically pathogenic variants in non-collagen AR OI genes primarily correspond to the classical collagen-related clinical types (OI 1–4) [29] (Table 2). Nevertheless, some differences in the details between non-collagen OI phenotypes may occur [18]. Whereas the homozygous and compound heterozygous variants of the *COL1A2* and other AR genes may result in severe phenotypes, individuals with the same heterozygous variants tend to be mildly affected or even asymptomatic [93–97]. Whilst the vast majority of lethal OI is associated with AD and AR variants in the collagen I genes, approximately 5% of lethal OI cases are caused by the *CRTAP*, *P3H1*, *PPIB* and *CREB3L1* pathogenic variants (genetic OI types VII-IX, XVI) [34, 83, 97–99]. The X-linked recessive (XLR) OI is associated with only two genes: *MBTPS2* (OMIM 300294) and *PLS3* (OMIM 300131). Currently, only 15 families with non-lethal XLR OI have been identified worldwide [100, 101].

#### Family planning for people without Osteogenesis Imperfecta

Besides those individuals personally affected by OI, reproductive challenges are also faced by members of families with an OI risk. This includes parents with a previous child affected by OI, parents of an unborn child with ultrasound findings of skeletal dysplasia and carriers of OI-associated pathogenic variants. All these individuals face a direct risk of having an offspring affected by OI. Some individuals face a higher probability of being OI carriers, such as consanguineous couples and members of founder communities in which a particular recessive variant is prevalent.

##### Preconception carrier screening

Preconception carrier screening (PCS) is conducted prior to pregnancy in couples with a risk of carrying a recessive OI variant (Fig. 2). As with genetic testing, PCS aims to identify couples at risk before pregnancy and provide them with genetic counselling about reproductive options, in order to support them in making an autonomous reproductive decision [102]. PCS is a general population screen for pathogenic variants conferring severe recessive disorders, and is recommended as an efficient means of minimizing the incidence of these disorders [102, 103]. PCS generally tests for several genetic disorders concurrently. The list of selected disorders varies between PCS providers, however the general criteria for inclusion onto a PCS panel includes relatively high incidence, shortened lifespan, severe mental or physical disability, and unavailability of treatment [104, 105]. OI is not commonly included in basic PCS due to its low incidence.

PCS programs differ in their pan-ethnic approach (which aims to screen carriers across different populations) and their population-specific approach (based on the screening of individuals from the same high-risk population). Pan-ethnic PCS is used for the screening of cystic fibrosis in individuals with European ancestry, whereas population-specific PCS is used for the screening of β-thalassemia in individuals from Mediterranean regions, and when testing for Tay-Sachs disease among Ashkenazi Jewish populations [106–108]. As OI has a lower incidence and higher genetic heterogeneity than these conditions, a population-specific approach may be relevant for the evaluation of OI risk in some populations. One example of a successful population-specific OI PCS is that reported by Mathijssen et al., in which the presence of the *CRTAP* frameshift variant was tested for in a Dutch founder population. The lethal AR OI variant was found to be carried by 4.1% of the genetic isolate village population, whereas the overall allele frequency in the general Dutch population is < 0.2% [109]. Another lethal AR OI variant in the *P3H1* gene was found to be carried in 1.5% of a West African study population and in 0.4% of a US African American study population, which is significantly higher than in the general population [90, 110]. Other OI founder variants have also been observed in Palestinian (*TMEM38B, FKBP10*), Israeli Arab Bedouin (*TMEM38B*), Hmong people (*WNT1*), German (*SEC24D, FKBP10*), and First Nations of Canada (*CRTAP*) study populations [77, 86, 88, 90, 91, 111–114]. The results of the Dutch PCS are very promising, however further accumulation of knowledge about OI genetic epidemiology is needed for development of OI population-specific PCS tests. Currently, only a limited number of founder populations benefit from a population-specific approach to OI PCS.

There are two main PCS methodologies: hybridization techniques, which concentrate on the detection of the most common targeted variants; and NGS, which is used to identify all variants in a panel of genes [115, 116]. Hybridization techniques allow the identification of a pathogenic variant via the complementary attachment of a labelled DNA probe to a target sequence. Hybridization techniques are an inexpensive and robust way to discover disorders in which only a limited number of causative genetic variants exists. In contrast, NGS is a more expensive method, yet it is better suited to the characteristics of the OI genetic heterogeneity and the individual nature of OI variants. NGS is therefore the most effective method for screening potential OI carriers.

OI screening is included in several currently available commercial extended PCS panels, since some OI forms (e.g. OI 2) are severely lifespan limiting. For example, the Inheritest® 500 PLUS Panel from Integrated Genetics (U.S.) includes nine AR OI genes (*BMP1, CRTAP, FKBP10, P3H1, PLOD2, PPIB, SERPINF1, TMEM38B* and *WNT1*), however, no screening of the *COL1A1, COL1A2* genes is provided. Collagen I genes are mainly associated with AD OI. However, AD and AR collagen I variants together account for 95% of lethal OI cases [67]. OI PCS is also provided by Asper Biogene (Estonia) via the Reprogenetics NGS panel. The panel includes 550 genes, amongst which are the *COL1A1, COL1A2, CRTAP* and *P3H1* genes, which significantly increases the sensitivity of this PCS panel with respect to lethal OI.

OI is genetically and phenotypically complex. In approximately 10% of individuals with mild OI, fractures (the main symptom of OI) may be absent [117]. The rarity of the OI disorder, in combination with a mild or asymptomatic phenotype, may increase the likelihood of it remaining undiagnosed, especially for mosaic individuals [118–120]. The inclusion of the *COL1A1* and *COL1A2* genes as well as AR genes in a PCS panel may help to prevent the transmission of a heterozygous variant from asymptomatic or mosaic parents to their offspring. It may also expand the diagnostic scope for OI in the general population.

PCS does have some limitations. Firstly, PCS does not screen for one of the main severe OI occurrence sources - parental gonadal mosaicism [121]. Secondly, PCS cannot be used to evaluate the risk of a de novo OI variant in prospective offspring, precluding full guarantee of the disorder’s absence [102]. Thirdly, the diverse expression of phenotypes and the absence of rigorous genotype-phenotype correlations obscures PCS clinical validity [122]. The existence of both lethal and non-lethal OI cases caused by the same variant adds additional complexity to the prediction of the phenotype [62, 63, 123]. Other limitations with PCS relate to NGS methodology limitations, such as VUS variants, lack of genetic counselling and non-specific genetic findings. These limitations may cause challenges for prospective parents when making a reproductive decision.

### Fertilisation options

If risk of OI transmission in prospective parents is confirmed (i.e. both parents are carriers of the AR variant or one of the parents has AD OI) then there are several options other than natural conception which may be appropriate. For example, in vitro fertilisation (IVF) with non-carrier donor germ cells or embryo, or IVF with own germ cells and preimplantation genetic testing (PGT) of embryos (Table 3, Fig. 3).

#### In vitro fertilisation

The use of IVF techniques is increasing in reproductive medicine worldwide. IVF is a process of oocyte fertilisation with sperm which occurs outside the body. IVF began as a therapeutic approach for couples with pregnancy loss or infertility, but is now a widely used technique for fertile individuals at risk of monogenic disorders, including OI. The first step of IVF is for the woman to undergo an ovulation stimulation process (superovulation). Matured oocytes are then removed from her ovaries. The oocytes are fertilised with sperm in vitro*.* Between the second to sixth day of development, an embryo is transferred into the uterus. Despite early hesitations, the latest meta-analyses show that IVF is not associated with a drastic increase in the risk of birth defects or genetic alterations in new-borns, as compared with natural conception [124–127].

##### In vitro fertilisation with donor sperm cell or oocyte

Prospective parents faced with a risk of OI who are considering IVF may have the option of non-carrier donor oocytes (if the female partner is affected) or non-carrier donor sperm cells (if the male partner is affected). In either case, the child will not be genetically related to one or other of the parents. Gamete donation is considered to be a practical and successful technique, provided that the non-carrier status of the donor is tested beforehand [128]. This option is particularly beneficial for individuals who, in addition to OI, have reduced fertility or infertility because of other, unrelated reproductive health issues. A donor oocyte may additionally benefit women affected with OI, because the possible risks associated with superovulation for bone density and the cardiovascular system will be avoided [129].

##### In vitro fertilisation with donor embryo transfer

Another fertilisation option is the implantation of an unaffected donor embryo, known as a prenatal adoption. Prenatal adoption could be recommended as an option where both prospective parents are affected with OI, or suffer from infertility. IVF with a donor embryo is in some countries juridically simpler than postnatal adoption and allows for an early mother-child bond.

##### Preimplantation genetic testing for monogenic disorders

For prospective parents facing a risk of OI and who will only consider having genetically related children, preimplantation genetic testing for monogenic disorders (PGT-M) may be advisable. PGT-M is used to scan embryos for harbouring the monogenic pathogenic variants [130].

PGT-M is based on the IVF technique, with the additional step of blastocyst genetic testing for the OI disorder. During the IVF process, on the fifth day of embryo development, a blastocyst trophectoderm biopsy is performed and a few cells are taken for genetic analysis (Fig. 3). Blastocysts are then frozen and stored. Genetic analysis identifies those blastocysts without OI pathogenic variants, which may be transferred into the uterus during a subsequent frozen embryo transfer cycle. Where implantation is unsuccessful, another stored unaffected cryopreserved blastocyst may be used. If further stored blastocysts are unavailable, the procedure may be repeated.

Due to the limited amount of genetic material collected during the biopsy, its genetic analysis requires a polymerase chain reaction (PCR)- or a whole genome amplification (WGA)-based DNA amplification [131, 132]. Historically, PCR-based methods have been widely used for PGT-M, [133] including when testing for AD OI variants in *COL1A1* and *COL1A2* genes [4, 134]. However, PCR-based methods may suffer from allele drop out (ADO), which can result in misdiagnosis and the implantation of an affected embryo. To reduce ADO, amplification may be combined with a modified linkage analysis. Modified linkage analysis identifies ADO by confirming polymorphic markers at sites close to the causative pathogenic variant [135].

In addition to single gene mutation analysis, the use of WGA enables embryos to be tested for aneuploidy, by array comparative genomic hybridization (aCGH) or single nucleotide polymorphism (SNP) arrays [131]. Rechitsky et al. reported a successful PGT-M for OI using a combined aCGH approach for chromosomal aberrations together with a multiplex nested polymerase chain reaction for OI pathogenic variant. The study reported unaffected new-borns in two out of three tested families with AD (*COL1A1, COL1A2*) and AR OI (*PPIB*) variants [136, 137]. The popularity of both aCGH and SNP array methods is increasing, as they allow fast and comprehensive testing of all chromosome pairs for aneuploidy, therefore enabling the selection of only euploid embryos for transfer and the reduction of miscarriage and chromosomal syndrome risks in new-borns [138, 139].

The SNP-array approach is also the basis for a karyomapping technique which uses SNP haplotyping to map chromosome origin from parents. In this way, a monogenic disorder can be diagnosed even without the development of a custom-made test for a specific pathogenic variant [140]. Karyomapping is widely used for PGT-M, however there are important limitations to the approach. These include the necessity of blood samples from both parents and a close relative of known disorder status (which are not always available), the consanguinity of parents and the de novo nature of the variant (which is particularly common for severe OI). Nevertheless, the approach may be useful in the case of milder familial OI cases. For example, in the case of a paternal-derived variant, a single-sperm-based SNP haplotyping, based on linked marker analysis efficiency, has been shown for AD OI (*COL1A1*) PGT-M, as reported recently by Chen et al. [136, 141, 142]. Several private companies (Igenomix, ORM genomics, Superior A.R.T.) also offer NGS-based PGT-M tests for OI families.

As with natural conception, 50% of AD OI IVF embryos may be mutation-free and 50% may be affected. In the case of AR OI carriers, 25% of embryos will be unaffected, 25% will be affected, and the remainder will be asymptomatic carriers of a pathogenic variant, like the parents [143]. In general, both carrier and unaffected embryos may be transferred [144]. However, a more accurate and balanced approach in carrier embryo transfer in case of OI may be needed. Where heterozygote individuals lack skeletal phenotype, they may be considered as apparently asymptomatic. However, in combination with variable phenotype expressivity and parental mosaicism risk, it is difficult to predict the effect of the variant in the offspring. Therefore, deep phenotyping and accurate clinical and genetic diagnosis in a centre with OI expertise is highly recommended for prospective parents prior to IVF and PGT-M procedures. Moreover, accuracy of the PGT procedure is estimated as 95 to 99.5% even in leading PGT centres, which still leaves a small chance for false diagnostic results [145, 146].

In conclusion, every case needs an individual approach, and the selection of PGT-M methods must be based on an OI family history, family members’ availability, the inheritance pattern and any OI causative variant properties observed in a kindred.

### Prenatal testing

After impregnation, prenatal screening to confirm or rule out the presence of OI is recommended (Table 3, Fig. 4). Prenatal tests allow for both the efficacy of the PGT approach as well as the OI risks associated with natural conception to be evaluated. Early prenatal diagnosis of OI is beneficial, as it provides enough time for a pregnancy management decision. It also allows consideration of delivery management and early OI treatment directly after birth, or even antenatally, with the developing method of mesenchymal stem cell transplantation (BOOSTB4 trial) [23, 147].

#### Non-invasive screening methods

##### Ultrasound (20th week of gestation)

Sporadic severe and lethal OI are usually first discovered during ultrasound screening at the 20th week of gestation [29]. Mild OI (OI 1 and 4) is usually not detectable with ultrasound, as the affected foetus has neither bowing of the long bones, nor intrauterine fractures, whereas a foetus with severe OI might suffer from numerous fractures, severe bowing of the long bones and demineralization of the skeleton. Lethal OI (OI 2) and OI 3 are similarly characterised by numerous fractures, severe bowing of long bones, demineralisation and well-visualized intracranial structures. A foetus affected with extremely severe lethal OI might also exhibit shortening and severe crumpling of long bones, and either thin or thick continuously beaded ribs [29, 148]. Specialists often find it difficult to make a differential prenatal diagnosis between OI 2 and 3.

##### Non-invasive prenatal testing (NIPT) (seventh to tenth week of gestation)

NIPT is a modern technique for non-invasive prenatal testing which has increased in popularity as an early (seventh to tenth week of gestation) and safe alternative to invasive tests. NIPT uses circulating cell-free foetal DNA (ccffDNA) from maternal blood plasma for genetic testing of the foetus [149, 150] (Fig. 4). Originating from apoptotic trophoblastic cells, ccffDNA accounts for around 10% of the DNA in maternal blood from the fifth week of gestation and clears soon after delivery [151–153]. NIPT may therefore be used to detect OI in the first trimester of the pregnancy. However, confirmative ultrasound screening, followed by amniocentesis or chorionic villus sampling (CVS) are conducted during the second trimester, which could have a negative psychological effect on prospective parents [29, 154]. NIPT has proved its sensitivity and specificity, so the test is now routinely used for the detection of foetal trisomy 21 and other aneuploidies; it is also used for sex- and RhD-determination of the foetus [155, 156].

To perform NIPT, a peripheral blood sample from the pregnant woman is required. The blood plasma is isolated, followed by cell-free DNA extraction. Then an NGS mutational analysis is performed, to reveal the presence of any OI-causative variant in the foetus. Maternal and foetal DNAs in the mother’s blood plasma are differentiated using a combination of bioinformatics and biostatistics tools [154, 157, 158]. In the near future, the further development of technologies will allow not only de novo or paternal AD cases of single gene disorders to benefit from NIPT (as has been the case), but also those with maternal familial AD and AR pathogenic variants [149, 159].

All reported OI cases identified with NIPT have harboured *COL1A1* and *COL1A2* pathogenic variants, making these genes an essential part of the skeletal dysplasia NIPT panels [5, 6, 154, 158, 160]. Additionally, some commercially available NIPT platforms appear to include OI. For example, GeneSAFE™ (Italy) and Vistara single gene NIPT by Natera (U.S.) screen for 44 and 21 severe disorders, respectively. Both tests are recommended for parents with paternal age > 40 and include the *COL1A1* and *COL1A2* genes.

The main issues with NIPT occur in the presence of a placental mosaicism and vanishing twins which, although rare, may cause misdiagnosis [159]. Early weeks of gestation and high body mass index of the mother are also common limitations of the NIPT [157]. However, with the help of a high coverage targeted NGS, NIPT now allows simultaneous testing for aneuploidy, foetal fraction and monogenic disorder, and is able to overcome low foetal fraction [154].

Other NIPT limitations are similar to those of invasive prenatal and postnatal genetic testing for OI and are dependent on the genetic testing approach chosen for screening. If a severe or lethal form of OI is suspected, the NIPT results are confirmed with invasive prenatal testing and followed by postnatal genetic diagnosis [158].

#### Invasive prenatal screening

Positive results from non-invasive testing are usually followed by invasive prenatal testing, such as CVS, amniocentesis or cordocentesis (Fig. 4). All three of these techniques for foetal cell extraction allow more foetal DNA be harvested than is possible with NIPT and are followed with standard OI genetic testing analysis (targeted NGS panel, Sanger sequencing, WES). The risk of pregnancy loss if invasive methods are used is estimated to be between approximately 0.5–2% [161]. As with ultrasound, prenatal genetic testing attempts to differ between OI 2 and 3 may be unsuccessful, which can have dramatic consequences for prospective parents and their families.

##### Chorionic villus sampling (CVS)

CVS uses placental tissue for sampling of the foetal DNA. The analysis is performed at the 10th–12th week of gestation, which is earlier than amniocentesis and cordocentesis. CVS uses material of placental origin, however this is associated with a risk of misdiagnosis because of placental mosaicism. CVS also enables cells to be biochemically checked for abnormal collagen production, associated with the presence of OI or other collagen-related disorders [162]. There is an approximately 1% risk of miscarriage after CVS [163].

##### Amniocentesis

Amniocentesis is based on analysis of the foetal DNA from the amniotic fluid. The procedure is usually performed at the 15th–20th week of gestation. In contrast to NIPT and CVS, amniocentesis allows foetal DNA to be analysed directly, which avoids misdiagnosis related to placental mosaicism. Amniocentesis is also able to overcome some NIPT-related testing limitations, such as twin pregnancies. The amniocentesis analysis is also useful where intrauterine infections need to be analysed [164]. Due to the invasiveness of the procedure, an approximately 0.5–1% risk of miscarriage exists [161].

##### Cordocentesis

Cordocentesis is a blood sampling from the umbilical cord. The method is used for blood disease treatment in utero or when other prenatal tests are not useful. Cordocentesis is performed at 22nd–24th weeks of gestation and carries many associated risks and complications. Miscarriage risk for cordocentesis is the highest of all the invasive methods, at approximately 1–2%.

### Other options

Other options which may be considered by prospective parents with a risk of OI include refraining from pregnancy and adoption [122].

The benefits of adoption include the absence of risks associated with pregnancy, delivery and lactation for OI women. However, depending on the country of residence, adoption may be a long and difficult process, with certain limitations for disabled parents.

### Ethical concerns

#### General remarks on the ethics of reproductive genetic testing for Osteogenesis Imperfecta

Reproductive decision-making is a complex process both for people with OI as well as for those whose families are or might be affected. Ethically and psychologically stressful, it often involves ambivalence and requires deliberation upon potentially incompatible beliefs, feelings and desires (such as the avoidance of suffering, the moral value of embryos, a desire for children, and feelings of guilt) [165]. The role of professional counselling in setting out options and highlighting the implications of different reproductive alternatives is important. For example, in comparison to more directive genetic counselling, a shared decision-making approach may result in decreased decisional conflict and decisional regret scores for women who terminate their pregnancy [166]. Figure 5 provides a decision-making tree to help with this process. Many of the reproductive options for those affected by OI are characterised by inherent uncertainty around the interpretation of test results. Although informing patients of these uncertainties is likely to make deliberation more complex, at the same time it supports autonomous decision-making and contributes towards patient education.

The responsibility to prevent suffering is often keenly felt by prospective parents and motivates many to reach out to reproductive technologies [167, 168]. However, for others this responsibility may be conceptualized differently. For example, an individual’s religious beliefs might not encompass such choices, but may lead instead to the acceptance of an obligation to care for those affected by OI.

For many reasons, not everyone at risk of having a child affected by OI will opt for reproductive technology. For some, the termination of a pregnancy is not acceptable. Some do not see the disease as a limitation. Others hope for future treatments, opt for adoption, or decide against having (more) children [165]. Furthermore, not all testing options are available everywhere: for example, in several European countries PGT is strictly regulated and only available for specific illnesses, and often the testing is not publicly funded [169].

With respect to PGT legislation, there are three main approaches: approval for a set of specific conditions (e.g. OI types I-XIII in UK; OI in South Korea); approval for all hereditary disorders (US, Russia, Brazil) and approval without a specific set of conditions (Germany, Italy, Austria, The Netherlands, France) (Supplemental Table S1) [170–173]. In the latter case, approval for the PGT is reviewed case-by-case and agreement from the relevant ethical committee is needed. Case review may be based on possible risk, disorder penetration, existence of genotype-phenotype correlations, severity, lethality, and treatability of the disorder according to the state’s or professional organization’s guidelines. Thus, in some countries, non-lethal, mild, and moderate OI forms might not be eligible for PGT.

Common general ethical challenges associated with prenatal diagnostics pertain to the availability of adequate counselling (made more difficult by clinical providers having to maintain expertise in this fast moving field), access issues, the potential impact of attitudes towards disabled people, commercial interests, the proliferation of testing options for clinically less serious disorders and the overall tendency towards the increased medicalisation of pregnancy [174]. Given the potential seriousness of OI for QoL, prenatal diagnosis clearly has an important role in preventing suffering and supporting informed decision-making in family planning. In the context of genetic services, the interests and rights of individuals should not be subject to population health aims [175].

#### Ethical aspects of testing options for Osteogenesis Imperfecta

Given the high genetic heterogeneity of OI, counselling both prior to and after testing is highly recommended. Especially for prospective parents faced with a risk of OI, it is important to clearly communicate which variants have been tested for and what has been excluded, but also to discuss the inherent uncertainties involved in prenatal diagnostics.

PCS for those at risk of carrying a recessive OI variant supports autonomous decision making and enables choices (e.g. PGT) which may be psychologically and ethically more acceptable to some individuals than testing during an already existing pregnancy. Importantly though, PCS cannot identify de novo variants or identify parental gonadal mosaicism. Sometimes PCS testing might be associated with stigmatisation and anxiety because of carrier status [176]. Depending on cultural and religious traditions, this stigmatisation may be gender-specific, causing discrimination and reproductive restrictions for some women. In contrast, studies show that PCS as a routine screening has a positive effect, as it can minimise the pressure on an individual to make a reproductive decision in a short period (i.e. if made before pregnancy) [177].

Opting for PGT offers the possibility of an unaffected embryo and avoids the risk of a later diagnosis in prenatal testing. On the other hand, PGT may raise numerous ethical concerns for future parents with OI [178]. A major limitation of this option pertains to the challenging process of IVF itself and its relatively low success rates (generally around 20–30%).

Prenatal testing - the discovery of severe and lethal OI with ultrasound - is usually only possible in quite advanced pregnancy (20th week). After a positive confirmation through invasive prenatal testing, prospective parents then face psychologically and ethically difficult decisions (preparation for the birth of an affected child, late termination of pregnancy). Empathetic and sensitive communication with parents is therefore a crucial part of care-provision. It has also been argued that, given the uncertainties involved in interpretation of tests, the articulation of a prognosis as *lethal* or as *incompatible with life* is inappropriate: such vocabulary may create additional stress for parents and tends to limit the clinical treatment options [123, 179].

NIPT enables earlier detection of possible OI and is favoured by those women who prefer less invasive methods. Earlier termination of pregnancy (should that be chosen) may be psychologically, emotionally and medically less stressful than later termination [174, 180]. NIPT may also be a more attractive option than PGT because it allows for natural conception, thus avoiding the demanding process of IVF, although NIPT is an alternative for those who consider termination of pregnancy an option [181]. NIPT is also likely to result in fewer miscarriages if lower number of invasive procedures are consequently undertaken [175].

## Conclusions

Being a rare heterogeneous genetic disorder, OI raises many technical and ethical reproductive issues for both medical professionals and patients. There is no single universal “best reproductive option” for all OI families, and reproductive strategies need to be carefully evaluated and discussed in order to achieve the most satisfying autonomous reproductive decision for each specific family. Access to information about advances in reproductive options and patient education on reproductive approaches are therefore crucially important. Early family planning, starting with pre-pregnancy OI genetic testing or PCS might benefit prospective parents facing a risk of OI, as it maximises the availability of various fertilisation and prenatal testing options. When considering fertilisation options such as natural conception, IVF with donor cells or embryo, or PGT-M, the advantages and disadvantages of each method should be evaluated, with a special attention given to the wishes, interests, health needs and opportunities of the prospective parents. During prenatal testing, safe non-invasive techniques, such as NIPT and ultrasound should prevail. Prospective parents’ decisions regarding invasive prenatal testing methods such as CVS, amniocentesis and cordocentesis, which carry risks for the pregnancy, need to be made autonomously, but with support.

The majority of OI reproductive ethical concerns are common to other genetic disorders. Issues around reproductive techniques, as well as reproductive decision-making and its consequences are faced by many families with different rare disorders. The most challenging OI-specific issues include the inaccurate prediction of OI type and lethality, insufficient research on fertility in OI patients, soft tissue issues in OI pregnancy, and a lack of ethical studies relating to anxiety and stigmatisation around reproductive decisions. Further improvements in the understanding of OI pathophysiology and nature may help to avoid some of the existing ethical dilemmas and improve the reproductive confidence of people affected by OI.

## Supplementary information

**Additional file 1: Table S1.** Availability and legal regulations of reproductive techniques for families at risk of Osteogenesis Imperfecta across countries. § - Criteria of a disorder allowed for PGT-M is suggested by guidelines of professional organizations [172].

## Data Availability

Not applicable.

## Abbreviations

aCGH: Array comparative genomic hybridization
AD: Autosomal dominant
ADO: Allele drop out
AR: Autosomal recessive
ccffDNA: Circulating cell-free foetal DNA
CVS: Chorionic villus sampling
IVF: In vitro fertilisation
LoF: Loss-of-function
MLPA: Multiplex ligation-dependent probe amplification
NGS: Next generation sequencing
NIPT: Non-invasive prenatal testing
OI: Osteogenesis Imperfecta
OMIM: Online Mendelian Inheritance in Man
PCR: Polymerase chain reaction
PCS: Preconception carrier screening
PGT: Preimplantation genetic testing
PGT-M: PGT for monogenic disease
QoL: Quality of life
SNP: Single nucleotide polymorphism
VUS: Variant of unknown significance
WES: Whole exome sequencing
WGA: Whole genome amplification
WGS: Whole genome sequencing
XLR: X-linked recessive

## References

[1] Chao L (2000). The meaning of life. Bioscience.

[2] Aduloju OP, Olaogun OD, Aduloju T (2018). Quality of life in women of reproductive age: a comparative study of infertile and fertile women in a Nigerian tertiary centre. J Obstet Gynaecol (Lahore).

[3] Geraedts J (2017). Healthy children without fear. EMBO Rep.

[4] De Vos A, Sermon K, Van De Velde H, Joris H, Vandervorst M, Lissens W (2000). Two pregnancies after preimplantation genetic diagnosis for osteogenesis imperfecta type I and type IV. Hum Genet.

[5] Yin X, Du Y, Zhang H, Wang Z, Wang J, Fu X (2018). Identification of a de novo fetal variant in osteogenesis imperfecta by targeted sequencing-based noninvasive prenatal testing. J Hum Genet.

[6] Mohan P, Parmar S, Saucier J, Jelsema R, Eaves K, Thomsen A (2019). 912: skeletal dysplasias screening by NIPT for single-gene disorders: clinical value of narrowing the differential diagnosis. Am J Obstet Gynecol.

[7] Mathijssen IB, Henneman L, van Eeten-Nijman JMC, Lakeman P, Ottenheim CPE, Redeker EJW (2015). Targeted carrier screening for four recessive disorders: high detection rate within a founder population. Eur J Med Genet.

[8] Byers PH, Steiner RD (1992). Osteogenesis imperfecta. Annu Rev Med.

[9] Sillence DO, Rimoin DL, Danks DM (1979). Clinical variability in osteogenesis imperfecta-variable expressivity or genetic heterogeneity. Birth Defects Orig Artic Ser.

[10] Kuurila K, Johansson R, Kaitila I, Grénman R (2002). Hearing loss in Finnish adults with Osteogenesis Imperfecta: a nationwide survey. Ann Otol Rhinol Laryngol.

[11] Lindahl K, Åström E, Rubin C-J, Grigelioniene G, Malmgren B, Ljunggren Ö (2015). Genetic epidemiology, prevalence, and genotype–phenotype correlations in the Swedish population with osteogenesis imperfecta. Eur J Hum Genet.

[12] Roughley PJ, Rauch F, Glorieux FH (2003). Osteogenesis imperfecta--clinical and molecular diversity. Eur Cell Mater.

[13] Buckwalter JA, Cooper RR (1987). Bone structure and function. Instr Course Lect.

[14] Daley E, Streeten EA, Sorkin JD, Kuznetsova N, Shapses SA, Carleton SM (2010). Variable bone fragility associated with an Amish COL1A2 variant and a knock-in mouse model. J Bone Miner Res.

[15] Shapiro JR, Lietman C, Grover M, Lu JT, Nagamani SC, Dawson BC (2013). Phenotypic variability of osteogenesis imperfecta type V caused by an IFITM5 mutation. J Bone Miner Res.

[16] Basel D, Steiner RD (2009). Osteogenesis imperfecta: recent findings shed new light on this once well-understood condition. Genet Med.

[17] Dwan K, Phillipi CA, Steiner RD, Basel D, Basel D (2014). Bisphosphonate therapy for osteogenesis imperfecta. Cochrane Database Syst. Rev.

[18] Marini JC, Forlino A, Bächinger HP, Bishop NJ, Byers PH, De Paepe A (2017). Osteogenesis imperfecta. Nat Rev Dis Prim.

[19] Marr C, Seasman A, Bishop N (2017). Managing the patient with osteogenesis imperfecta: a multidisciplinary approach. J Multidiscip Healthc.

[20] Monti E, Mottes M, Fraschini P, Brunelli P, Forlino A, Venturi G (2010). Current and emerging treatments for the management of osteogenesis imperfecta. Ther Clin Risk Manag.

[21] Rijks EBG, Bongers BC, Vlemmix MJG, Boot AM, Van Dijk ATH, Sakkers RJB (2015). Efficacy and safety of bisphosphonate therapy in children with osteogenesis imperfecta: a systematic review. Horm Res Paediatr.

[22] Semler O, Netzer C, Hoyer-Kuhn H, Becker J, Eysel P, Schoenau E (2012). First use of the RANKL antibody denosumab in osteogenesis imperfecta type VI. J Musculoskelet Neuronal Interact.

[23] Hill M, Lewis C, Riddington M, Crowe B, DeVile C, David AL (2019). Stakeholder views and attitudes towards prenatal and postnatal transplantation of fetal mesenchymal stem cells to treat Osteogenesis Imperfecta. Eur J Hum Genet.

[24] Besio R, Iula G, Garibaldi N, Cipolla L, Sabbioneda S, Biggiogera M (2018). 4-PBA ameliorates cellular homeostasis in fibroblasts from osteogenesis imperfecta patients by enhancing autophagy and stimulating protein secretion. Biochim Biophys Acta Mol Basis Dis.

[25] Widmann RF, Laplaza FJ, Bitan FD, Brooks CE, Root L (2002). Quality of life in osteogenesis imperfecta. Int Orthop.

[26] Dahan-Oliel N, Oliel S, Tsimicalis A, Montpetit K, Rauch F, Dogba MJ (2016). Quality of life in osteogenesis imperfecta: a mixed-methods systematic review. Am J Med Genet A.

[27] Hill CL, Baird WO, Walters SJ (2014). Quality of life in children and adolescents with Osteogenesis Imperfecta: a qualitative interview based study. Health Qual Life Outcomes.

[28] Folkestad L, Hald JD, Canudas-Romo V, Gram J, Hermann AP, Langdahl B (2016). Mortality and causes of death in patients with Osteogenesis Imperfecta: a register-based nationwide cohort study. J Bone Miner Res.

[29] Van Dijk FS, Sillence DO (2014). Osteogenesis imperfecta: clinical diagnosis, nomenclature and severity assessment. Am J Med Genet A.

[30] Sillence DO, Barlow KK, Garber AP, Hall JG, Rimoin DL (1984). Osteogenesis imperfecta type II delineation of the phenotype with reference to genetic heterogeneity. Am J Med Genet.

[31] Glorieux FH, Rauch F, Plotkin H, Ward L, Travers R, Roughley P (2000). Type V Osteogenesis Imperfecta: a new form of brittle bone disease. J Bone Miner Res.

[32] Patel RM, Nagamani SCS, Cuthbertson D, Campeau PM, Krischer JP, Shapiro JR (2015). A cross-sectional multicenter study of osteogenesis imperfecta in North America - results from the linked clinical research centers. Clin Genet.

[33] Zhytnik L, Maasalu K, Duy BH, Pashenko A, Khmyzov S, Reimann E (2019). IFITM5 pathogenic variant causes osteogenesis imperfecta V with various phenotype severity in Ukrainian and Vietnamese patients. Hum Genomics.

[34] Bardai G, Moffatt P, Glorieux FH, Rauch F (2016). DNA sequence analysis in 598 individuals with a clinical diagnosis of osteogenesis imperfecta: diagnostic yield and mutation spectrum. Osteoporos Int.

[35] Sillence DO, Senn A, Danks DM (1979). Genetic heterogeneity in osteogenesis imperfecta. J Med Genet.

[36] Zhytnik L, Maasalu K, Reimann E, Prans E, Kõks S, Märtson A (2017). Mutational analysis of COL1A1 and COL1A2 genes among Estonian osteogenesis imperfecta patients. Hum Genomics.

[37] Maioli Margherita, Gnoli Maria, Boarini Manila, Tremosini Morena, Zambrano Anna, Pedrini Elena, Mordenti Marina, Corsini Serena, D’Eufemia Patrizia, Versacci Paolo, Celli Mauro, Sangiorgi Luca (2019). Genotype–phenotype correlation study in 364 osteogenesis imperfecta Italian patients. European Journal of Human Genetics.

[38] Marini JC, Forlino A, Cabral WA, Barnes AM, San Antonio JD, Milgrom S (2007). Consortium for osteogenesis imperfecta mutations in the helical domain of type I collagen: regions rich in lethal mutations align with collagen binding sites for integrins and proteoglycans. Hum Mutat.

[39] Leikin EM, S. Collagen Structure, Folding and Function (2014). Osteogenes. Imperfecta.

[40] Eyre DR, Paz MA, Gallop PM (1984). Cross-linking in collagen and elastin. Annu Rev Biochem.

[41] Shoulders MD, Raines RT (2009). Collagen structure and stability. Annu Rev Biochem.

[42] Forlino A, Cabral WA, Barnes AM, Marini JC (2011). New perspectives on osteogenesis imperfecta. Nat Rev Endocrinol.

[43] Dubail J, Brunelle P, Baujat G, Huber C, Doyard M, Michot C, et al. Homozygous loss-of-function mutations in CCDC134 are responsible for a severe form of osteogenesis imperfecta. J Bone Miner Res. 2020. 10.1002/jbmr.4011. [Epub ahead of print]. 10.1002/jbmr.4011 32181939

[44] Cozzolino M, Perelli F, Maggio L, Coccia ME, Quaranta M, Gizzo S (2016). Management of osteogenesis imperfecta type I in pregnancy; a review of literature applied to clinical practice. Arch Gynecol Obstet.

[45] Chamunyonga F, Masendeke KL, Mateveke B (2019). Osteogenesis imperfecta and pregnancy: a case report. J Med Case Rep.

[46] Ghiossi CE, Goldberg JD, Haque IS, Lazarin GA, Wong KK (2018). Clinical utility of expanded carrier screening: reproductive behaviors of at-risk couples. J Genet Couns.

[47] Zhytnik L, Maasalu K, Duy BH, Pashenko A, Khmyzov S, Reimann E (2019). *De novo* and inherited pathogenic variants in collagen-related osteogenesis imperfecta. Mol Genet Genomic Med.

[48] The Voice of People with OI | OIFE - Osteogenesis Imperfecta Federation Europe. Available from: https://oife.org/2018/04/02/the-voice-of-people-with-oi/. Cited 22 Oct 2019.

[49] Byers PH, Krakow D, Nunes ME, Pepin M (2006). Genetic evaluation of suspected osteogenesis imperfecta (OI). Genet Med.

[50] van Dijk FS, Byers PH, Dalgleish R, Malfait F, Maugeri A, Rohrbach M (2012). EMQN best practice guidelines for the laboratory diagnosis of osteogenesis imperfecta. Eur J Hum Genet.

[51] Mrosk Julia, Bhavani Gandham SriLakshmi, Shah Hitesh, Hecht Jochen, Krüger Ulrike, Shukla Anju, Kornak Uwe, Girisha Katta Mohan (2018). Diagnostic strategies and genotype-phenotype correlation in a large Indian cohort of osteogenesis imperfecta. Bone.

[52] Pandya NK, Baldwin K, Kamath AF, Wenger DR, Hosalkar HS (2011). Unexplained fractures: child abuse or bone disease? A systematic review. Clin Orthop Relat Res.

[53] Cho T-J, Lee K-E, Lee S-K, Song SJ, Kim KJ, Jeon D (2012). A single recurrent mutation in the 5′-UTR of IFITM5 causes Osteogenesis Imperfecta type V. Am J Hum Genet.

[54] Semler O, Garbes L, Keupp K, Swan D, Zimmermann K, Becker J (2012). A mutation in the 5′-UTR of IFITM5 creates an in-frame start codon and causes autosomal-dominant Osteogenesis Imperfecta type V with hyperplastic callus. Am J Hum Genet.

[55] Dalgleish R (1997). The human type I collagen mutation database. Nucleic Acids Res.

[56] Shapiro JR, Lietman C, Grover M, Lu JT, Nagamani SC, Dawson BC (2013). Phenotypic variability of Osteogenesis Imperfecta type V caused by an *IFITM 5* mutation. J Bone Miner Res.

[57] Gistelinck C, Kwon RY, Malfait F, Symoens S, Harris MP, Henke K (2018). Zebrafish type I collagen mutants faithfully recapitulate human type I collagenopathies. Proc Natl Acad Sci U S A.

[58] Prockop DJ, Kivirikko KI (1984). Heritable diseases of collagen. N Engl J Med.

[59] Forlino A, Marini JC (2000). Osteogenesis Imperfecta: prospects for molecular therapeutics. Mol Genet Metab.

[60] Ben Amor IM, Glorieux FH, Rauch F (2011). Genotype-phenotype correlations in autosomal dominant osteogenesis imperfecta. J Osteoporos.

[61] Zhytnik L, Maasalu K, Pashenko A, Khmyzov S, Reimann E, Prans E (2019). COL1A1/2 pathogenic variants and phenotype characteristics in Ukrainian Osteogenesis Imperfecta patients. Front Genet.

[62] Bodian DL, Madhan B, Brodsky B, Klein TE (2008). Predicting the clinical lethality of osteogenesis imperfecta from collagen glycine mutations. Biochemistry.

[63] Alhousseini A, Mahaseth M, Zeineddine S, Jaiman S, Berman S, Bryant D (2019). A non-lethal Osteogenesis Imperfecta type II mutation. Gynecol Obstet Investig.

[64] Rauch F, Lalic L, Roughley P, Glorieux FH (2010). Genotype-phenotype correlations in nonlethal osteogenesis imperfecta caused by mutations in the helical domain of collagen type I. Eur J Hum Genet.

[65] Steiner RD, Adsit J, Basel D (1993). COL1A1/2-Related Osteogenesis Imperfecta. GeneReviews®.

[66] Risch N, Reich EW, Wishnick MM, McCarthy JG (1987). Spontaneous mutation and parental age in human. Am J Hum Genet.

[67] Pyott SM, Pepin MG, Schwarze U, Yang K, Smith G, Byers PH (2011). Recurrence of perinatal lethal osteogenesis imperfecta in sibships: parsing the risk between parental mosaicism for dominant mutations and autosomal recessive inheritance. Genet Med.

[68] Frederiksen AL, Duno M, Johnsen IBG, Nielsen MF, Krøigård AB (2016). Asymptomatic parental mosaicism for osteogenesis imperfecta associated with a new splice site mutation in COL1A2. Clin Case Rep.

[69] Chen C-P, Lin S-P, Su Y-N, Chern S-R, Su J-W, Wang W (2013). Prenatal diagnosis of recurrent autosomal dominant osteogenesis imperfecta associated with unaffected parents and paternal gonadal mosaicism. Taiwan J Obstet Gynecol.

[70] Marini JC, Reich A, Smith SM (2014). Osteogenesis imperfecta due to mutations in non-collagenous genes: lessons in the biology of bone formation. Curr Opin Pediatr.

[71] Morello R, Bertin TK, Chen Y, Hicks J, Tonachini L, Monticone M (2006). CRTAP is required for Prolyl 3- hydroxylation and mutations cause recessive Osteogenesis Imperfecta. Cell.

[72] van Dijk FS, Nesbitt IM, Zwikstra EH, Nikkels PGJ, Piersma SR, Fratantoni SA (2009). PPIB mutations cause severe Osteogenesis Imperfecta. Am J Hum Genet.

[73] Cabral WA, Chang W, Barnes AM, Weis M, Scott MA, Leikin S (2007). Prolyl 3-hydroxylase 1 deficiency causes a recessive metabolic bone disorder resembling lethal/severe osteogenesis imperfecta. Nat Genet.

[74] Steinlein OK, Aichinger E, Trucks H, Sander T (2011). Mutations in FKBP10 can cause a severe form of isolated Osteogenesis imperfecta. BMC Med Genet.

[75] Christiansen HE, Schwarze U, Pyott SM, AlSwaid A, Al Balwi M, Alrasheed S (2010). Homozygosity for a missense mutation in SERPINH1, which encodes the collagen chaperone protein HSP47, results in severe recessive Osteogenesis Imperfecta. Am J Hum Genet.

[76] Asharani PV, Keupp K, Semler O, Wang W, Li Y, Thiele H (2012). Attenuated BMP1 function compromises osteogenesis, leading to bone fragility in humans and zebrafish. Am J Hum Genet.

[77] Garbes L, Kim K, Rieß A, Hoyer-Kuhn H, Beleggia F, Bevot A (2015). Mutations in SEC24D, encoding a component of the COPII machinery, cause a syndromic form of osteogenesis imperfecta. Am J Hum Genet.

[78] Balasubramanian M, Hurst J, Brown S, Bishop NJ, Arundel P, DeVile C (2017). Compound heterozygous variants in NBAS as a cause of atypical osteogenesis imperfecta. Bone.

[79] Ha-Vinh R, Alanay Y, Bank RA, Campos-Xavier AB, Zankl A, Superti-Furga A (2004). Phenotypic and molecular characterization of Bruck syndrome (Osteogenesis imperfecta with contractures of the large joints) caused by a recessive mutation in PLOD2. Am J Med Genet.

[80] Becker J, Semler O, Gilissen C, Li Y, Bolz HJ, Giunta C (2011). Exome sequencing identifies truncating mutations in human SERPINF1 in autosomal-recessive osteogenesis imperfecta. Am J Hum Genet.

[81] Doyard M, Bacrot S, Huber C, Di Rocco M, Goldenberg A, Aglan MS (2018). *FAM46A* mutations are responsible for autosomal recessive osteogenesis imperfecta. J Med Genet.

[82] Moosa S, Yamamoto GL, Garbes L, Keupp K, Beleza-Meireles A, Moreno CA (2019). Autosomal-recessive mutations in MESD cause Osteogenesis Imperfecta. Am J Hum Genet.

[83] Symoens S, Malfait F, D’hondt S, Callewaert B, Dheedene A, Steyaert W (2013). Deficiency for the ER-stress transducer OASIS causes severe recessive osteogenesis imperfecta in humans. Orphanet J Rare Dis.

[84] Mendoza-Londono R, Fahiminiya S, Majewski J, Tétreault M, Nadaf J, Kannu P (2015). Recessive Osteogenesis Imperfecta caused by missense mutations in SPARC. Am J Hum Genet.

[85] Shaheen R, Alazami AM, Alshammari MJ, Faqeih E, Alhashmi N, Mousa N (2012). Study of autosomal recessive osteogenesis imperfecta in Arabia reveals a novel locus defined by *TMEM38B* mutation. J Med Genet.

[86] Pyott SM, Tran TT, Leistritz DF, Pepin MG, Mendelsohn NJ, Temme RT (2013). WNT1 mutations in families affected by moderately severe and progressive recessive Osteogenesis Imperfecta. Am J Hum Genet.

[87] Lapunzina P, Aglan M, Temtamy S, Caparrós-Martín JA, Valencia M, Letón R (2010). Identification of a frameshift mutation in osterix in a patient with recessive Osteogenesis Imperfecta. Am J Hum Genet.

[88] Essawi O, Symoens S, Fannana M, Darwish M, Farraj M, Willaert A (2018). Genetic analysis of osteogenesis imperfecta in the Palestinian population: molecular screening of 49 affected families. Mol Genet Genomic Med.

[89] Willaert A, Malfait F, Symoens S, Gevaert K, Kayserili H, Megarbane A (2009). Recessive osteogenesis imperfecta caused by LEPRE1 mutations: clinical documentation and identification of the splice form responsible for prolyl 3-hydroxylation. J Med Genet.

[90] Cabral WA, Barnes AM, Adeyemo A, Cushing K, Chitayat D, Porter FD (2012). A founder mutation in LEPRE1 carried by 1.5% of West Africans and 0.4% of African Americans causes lethal recessive osteogenesis imperfecta. Genet Med.

[91] Ward LM, Rauch F, Travers R, Chabot G, Azouz EM, Lalic L (2002). Osteogenesis imperfecta type VII: an autosomal recessive form of brittle bone disease. Bone.

[92] Kurt-Sukur ED, Simsek-Kiper PO, Utine GE, Boduroglu K, Alanay Y (2015). Experience of a skeletal dysplasia registry in Turkey: a five-years retrospective analysis. Am J Med Genet A.

[93] Ackermann AM, Levine MA (2017). Compound heterozygous mutations in *COL1A1* associated with an atypical form of type I osteogenesis imperfecta. Am J Med Genet A.

[94] De Paepe A, Nuytinck L, Raes M, Fryns JP (1997). Homozygosity by descent for a COL1A2 mutation in two sibs with severe osteogenesis imperfecta and mild clinical expression in the heterozygotes. Hum Genet.

[95] Pope FM, Nicholls AC, McPheat J, Talmud P, Owen R (1985). Collagen genes and proteins in osteogenesis imperfecta. J Med Genet.

[96] Keupp K, Beleggia F, Kayserili H, Barnes AM, Steiner M, Semler O (2013). Mutations in WNT1 cause different forms of bone fragility. Am J Hum Genet.

[97] Keller RB, Tran TT, Pyott SM, Pepin MG, Savarirayan R, McGillivray G (2018). Monoallelic and biallelic CREB3L1 variant causes mild and severe osteogenesis imperfecta, respectively. Genet Med.

[98] Bodian DL, Chan T-F, Poon A, Schwarze U, Yang K, Byers PH (2009). Mutation and polymorphism spectrum in osteogenesis imperfecta type II: implications for genotype-phenotype relationships. Hum Mol Genet.

[99] Pyott SM, Schwarze U, Christiansen HE, Pepin MG, Leistritz DF, Dineen R (2011). Mutations in PPIB (cyclophilin B) delay type I procollagen chain association and result in perinatal lethal to moderate osteogenesis imperfecta phenotypes. Hum Mol Genet.

[100] van Dijk FS, Zillikens MC, Micha D, Riessland M, Marcelis CLM, de Die-Smulders CE (2013). PLS3 mutations in X-linked osteoporosis with fractures. N Engl J Med.

[101] Lindert U, Cabral WA, Ausavarat S, Tongkobpetch S, Ludin K, Barnes AM (2016). MBTPS2 mutations cause defective regulated intramembrane proteolysis in X-linked osteogenesis imperfecta. Nat Commun.

[102] Antonarakis Stylianos E. (2019). Carrier screening for recessive disorders. Nature Reviews Genetics.

[103] Cao A, Kan YW (2013). The prevention of thalassemia. Cold Spring Harb Perspect Med.

[104] Chokoshvili D, Vears D, Borry P (2018). Expanded carrier screening for monogenic disorders: where are we now?. Prenat Diagn.

[105] Lazarin GA, Hawthorne F, Collins NS, Platt EA, Evans EA, Haque IS (2014). Systematic classification of disease severity for evaluation of expanded carrier screening panels. PLoS One.

[106] Kaback Michael M. (2000). Population-based genetic screening for reproductive counseling: the Tay-Sachs disease model. European Journal of Pediatrics.

[107] Cao A, Saba L, Galanello R, Rosatelli MC (1997). Molecular diagnosis and carrier screening for beta thalassemia. JAMA.

[108] American College of Obstetricians and Gynecologists Committee on Genetics. Committee Opinion No. 486: Update on Carrier Screening forCystic Fibrosis, Obstetrics & Gynecology. 2011;117(4):1028–31. 10.1097/AOG.0b013e31821922c2. 10.1097/AOG.0b013e31821922c2 21422883

[109] Mathijssen IB, Holtkamp KCA, Ottenheim CPE, Van Eeten-Nijman JMC, Lakeman P, Meijers-Heijboer H (2018). Preconception carrier screening for multiple disorders: evaluation of a screening offer in a Dutch founder population /631/208/2489/1512 /692/700/478/2772 article. Eur J Hum Genet.

[110] Pepin MG, Schwarze U, Singh V, Romana M, Jones-LeCointe A, Byers PH (2013). Allelic background of *LEPRE1* mutations that cause recessive forms of osteogenesis imperfecta in different populations. Mol Genet Genomic Med.

[111] Laine CM, Joeng KS, Campeau PM, Kiviranta R, Tarkkonen K, Grover M (2013). WNT1 mutations in early-onset osteoporosis and osteogenesis imperfecta. N Engl J Med.

[112] Volodarsky M, Markus B, Cohen I, Staretz-Chacham O, Flusser H, Landau D (2013). A deletion mutation in TMEM38B associated with autosomal recessive Osteogenesis Imperfecta. Hum Mutat.

[113] Alanay Y, Avaygan H, Camacho N, Utine GE, Boduroglu K, Aktas D (2010). Mutations in the gene encoding the RER protein FKBP65 cause autosomal-recessive Osteogenesis Imperfecta. Am J Hum Genet.

[114] Plantinga M, Birnie E, Abbott KM, Sinke RJ, Lucassen AM, Schuurmans J (2016). Population-based preconception carrier screening: how potential users from the general population view a test for 50 serious diseases. Eur J Hum Genet.

[115] Bell CJ, Dinwiddie DL, Miller NA, Hateley SL, Ganusova EE, Mudge J (2011). Carrier testing for severe childhood recessive diseases by next-generation sequencing. Sci Transl Med.

[116] Srinivasan BS, Evans EA, Flannick J, Patterson AS, Chang CC, Pham T (2010). A universal carrier test for the long tail of Mendelian disease. Reprod BioMed Online.

[117] Deodhar AA, Woolf AD (2000). Fragile without fractures. Ann Rheum Dis.

[118] Binh HD, Maasalu K, Dung VC, Ngoc CTB, Hung TT, Nam TV (2017). The clinical features of osteogenesis imperfecta in Vietnam. Int Orthop.

[119] Parikh JH, Skreydel M, Mozlin R. Diving deeper into the blue: a case of osteogenesis imperfecta with ocular involvement undiagnosed for 25 years. American Academy of Optometry. 2017. Abstract.

[120] Rebelo M, Lima J, Vieira JD, Costa JN (2011). An unusual presentation of osteogenesis imperfecta type I. Int Med Case Rep J.

[121] Campbell IM, Yuan B, Robberecht C, Pfundt R, Szafranski P, McEntagart ME (2014). Parental somatic mosaicism is underrecognized and influences recurrence risk of genomic disorders. Am J Hum Genet.

[122] Henneman L, Borry P, Chokoshvili D, Cornel MC, Van El CG, Forzano F (2016). Responsible implementation of expanded carrier screening. Eur J Hum Genet.

[123] Kidszun A, Linebarger J, Walter JK, Paul NW, Fruth A, Mildenberger E (2016). What if the prenatal diagnosis of a lethal anomaly turns out to be wrong?. Pediatrics.

[124] Qin JB, Sheng XQ, Wang H, Chen GC, Yang J, Yu H (2017). Worldwide prevalence of adverse pregnancy outcomes associated with in vitro fertilization/intracytoplasmic sperm injection among multiple births: a systematic review and meta-analysis based on cohort studies. Arch Gynecol Obstet.

[125] Davies M, Rumbold A, Marino J, Willson K, Giles L, Whitrow M (2017). Maternal factors and the risk of birth defects after IVF and ICSI: a whole of population cohort study. BJOG An Int J Obstet Gynaecol.

[126] Banker M, Arora P, Banker J, Benani H, Shah S, Lalitkumar PGL (2019). Prevalence of structural birth defects in IVF-ICSI pregnancies resulting from autologous and donor oocytes in Indian sub-continent: results from 2444 births. Acta Obstet Gynecol Scand.

[127] Verlinsky Yury (1996). Preimplantation genetic diagnosis. Journal of Assisted Reproduction and Genetics.

[128] Oocyte donation - Fertility - NCBI Bookshelf. Available from: https://www.ncbi.nlm.nih.gov/books/NBK327787/. Cited 9 Dec 2019.

[129] Zhang J, Lai Z, Shi L, Tian Y, Luo A, Xu Z (2018). Repeated superovulation increases the risk of osteoporosis and cardiovascular diseases by accelerating ovarian aging in mice. Aging (Albany. NY).

[130] Verlinsky Yury (1996). Preimplantation genetic diagnosis. Journal of Assisted Reproduction and Genetics.

[131] Treff NR, Zimmerman RS (2017). Advances in preimplantation genetic testing for monogenic disease and aneuploidy. Annu Rev Genomics Hum Genet.

[132] Treff NR, Zimmerman R, Bechor E, Hsu J, Rana B, Jensen J (2019). Validation of concurrent preimplantation genetic testing for polygenic and monogenic disorders, structural rearrangements, and whole and segmental chromosome aneuploidy with a single universal platform. Eur J Med Genet.

[133] Sullivan-Pyke C, Dokras A (2018). Preimplantation genetic screening and preimplantation genetic diagnosis. Obstet Gynecol Clin North Am.

[134] Jee Kim M, Lee H-S, Won Choi H, Kyu Lim C, Won Cho J, Young Kim J (2008). Establishment and application of molecular genetic techniques for preimplantation genetic diagnosis of Osteogenesis Imperfecta. Korean J Reprod Med.

[135] Piyamongkol W (2003). Detailed investigation of factors influencing amplification efficiency and allele drop-out in single cell PCR: implications for preimplantation genetic diagnosis. Mol Hum Reprod.

[136] Kuliev A, Rechitsky S (2017). Preimplantation genetic testing: current challenges and future prospects. Expert Rev Mol Diagn.

[137] Rechitsky S, Pakhalchuk T, San Ramos G, Goodman A, Zlatopolsky Z, Kuliev A (2015). First systematic experience of preimplantation genetic diagnosis for single-gene disorders, and/or preimplantation human leukocyte antigen typing, combined with 24-chromosome aneuploidy testing. Fertil Steril.

[138] Brezina PR, Ke RW, Kutteh WH (2013). Preimplantation genetic screening: a practical guide. Clin Med Insights Reprod Health.

[139] Schaaf C, Scott D, Wiszniewska J, Beaudet A (2011). Identification of incestuous parental relationships by SNP-based DNA microarrays. Lancet.

[140] Natesan SA, Bladon AJ, Coskun S, Qubbaj W, Prates R, Munne S (2014). Genome-wide karyomapping accurately identifies the inheritance of single-gene defects in human preimplantation embryos in vitro. Genet Med.

[141] Chen L, Diao Z, Xu Z, Zhou J, Yan G, Sun H (2019). The clinical application of single-sperm-based SNP haplotyping for PGD of osteogenesis imperfecta. Syst Biol Reprod Med.

[142] Renwick PJ, Trussler J, Ostad-Saffari E, Fassihi H, Black C, Braude P (2006). Proof of principle and first cases using preimplantation genetic haplotyping - a paradigm shift for embryo diagnosis. Reprod Biomed Online.

[143] Harper JC, Sengupta SB (2012). Preimplantation genetic diagnosis: State of the ART 2011. Hum Genet.

[144] Zanetti BF, Braga DPDAF, Azevedo MDC, Setti AS, Figueira RCS, Iaconelli A (2019). Preimplantation genetic testing for monogenic diseases: a Brazilian IVF centre experience. J Bras Reprod Assist.

[145] Brezina PR, Kutteh WH, Bailey AP, Ke RW (2016). Preimplantation genetic screening (PGS) is an excellent tool, but not perfect: a guide to counseling patients considering PGS. Fertil Steril.

[146] Kuliev A, Verlinsky O, Rechitsky S (2013). Safety, accuracy and reproductive outcome of preimplantation genetic diagnosis.

[147] Sagar R, Walther-Jallow L, David AL, Götherström C, Westgren M (2018). Fetal mesenchymal stromal cells: an opportunity for prenatal cellular therapy. Curr Stem Cell Rep.

[148] Sillence DO (1988). Osteogenesis Imperfecta nosology and genetics. Ann N Y Acad Sci.

[149] Bustamante-Aragonés A, Rodríguez de Alba M, Perlado S, Trujillo-Tiebas MJ, Arranz JP, Díaz-Recasens J (2012). Non-invasive prenatal diagnosis of single-gene disorders from maternal blood. Gene.

[150] Lun FMF, Chiu RWK, Chan KCA, Tak YL, Tze KL, Lo YMD (2008). Microfluidics digital PCR reveals a higher than expected fraction of fetal DNA in maternal plasma. Clin Chem.

[151] Alberry M, Maddocks D, Jones M, Abdel Hadi M, Abdel-Fattah S, Avent N (2007). Free fetal DNA in maternal plasma in anembryonic pregnancies: confirmation that the origin is the trophoblast. Prenat Diagn.

[152] Chan KCA, Zhang J, Hui ABY, Wong N, Lau TK, Leung TN (2004). Size distributions of maternal and fetal DNA in maternal plasma. Clin Chem.

[153] Guibert J, Benachi A, Grebille AG, Ernault P, Zorn JR, Costa JM (2003). Kinetics of SRY gene appearance in maternal serum: detection by real time PCR in early pregnancy after assisted reproductive technique. Hum Reprod.

[154] Malcher Carolina, Yamamoto Guilherme L., Burnham Philip, Ezquina Suzana A.M., Lourenço Naila C.V., Balkassmi Sahilla, Antonio David S. Marco, Hsia Gabriella S.P., Gollop Thomaz, Pavanello Rita C., Lopes Marco Antonio, Bakker Egbert, Zatz Mayana, Bertola Débora, Vlaminck Iwijn De, Passos-Bueno Maria Rita (2018). Development of a comprehensive noninvasive prenatal test. Genetics and Molecular Biology.

[155] Koumbaris G, Kypri E, Tsangaras K, Achilleos A, Mina P, Neofytou M (2016). Cell-Free DNA analysis of targeted genomic regions in maternal plasma for non-invasive prenatal testing of trisomy 21, trisomy 18, trisomy 13, and fetal sex. Clin Chem.

[156] Žilina O, Rekker K, Kaplinski L, Sauk M, Paluoja P, Teder H (2019). Creating basis for introducing non-invasive prenatal testing in the Estonian public health setting. Prenat Diagn.

[157] Teder H, Paluoja P, Rekker K, Salumets A, Krjutškov K, Palta P (2019). Computational framework for targeted high-coverage sequencing based NIPT. PLoS One.

[158] Zhang J, Li J, Saucier JB, Feng Y, Jiang Y, Sinson J (2019). Non-invasive prenatal sequencing for multiple Mendelian monogenic disorders using circulating cell-free fetal DNA. Nat Med.

[159] Breveglieri G, D’Aversa E, Finotti A, Borgatti M (2019). Non-invasive prenatal testing using fetal DNA. Mol Diagnosis Ther.

[160] Dan S, Yuan Y, Wang Y, Chen C, Gao C, Yu S (2016). Non-invasive prenatal diagnosis of lethal skeletal dysplasia by targeted capture sequencing of maternal plasma. PLoS One.

[161] Kozlowski P, Knippel A, Stressig R (2008). Individual risk of fetal loss following routine second trimester amniocentesis: a controlled study of 20 460 cases. Ultraschall der Medizin.

[162] Raghunath M, Steinmann B, Delozier-Blanchet C, Extermann P, Superti-Furga A (1994). Prenatal diagnosis of collagen disorders by direct biochemical analysis of chorionic villus biopsies. Pediatr Res.

[163] Alfirevic Z, Navaratnam K, Mujezinovic F. Amniocentesis and chorionic villus sampling for prenatal diagnosis. Cochrane Database Syst Rev. 2017;9(9):CD003252. Published 2017 Sep 4. 10.1002/14651858.CD003252.pub2. 10.1002/14651858.CD003252.pub2 PMC6483702 28869276

[164] Connolly KA (2016). Amniocentesis: a contemporary review. World J Obstet Gynecol.

[165] Decruyenaere M, Evers-Kiebooms G, Boogaerts A, Philippe K, Demyttenaere K, Dom R (2007). The complexity of reproductive decision-making in asymptomatic carriers of the Huntington mutation. Eur J Hum Genet.

[166] Moudi Z, Phanodi Z, Ansari H, Zohour MM (2018). Decisional conflict and regret: shared decision-making about pregnancy affected by β-thalassemia major in southeast of Iran. J Hum Genet.

[167] Downing C (2005). Negotiating responsibility: case studies of reproductive decision-making and prenatal genetic testing in families facing Huntington disease. J Genet Couns.

[168] Kalfoglou AL, Doksum T, Bernhardt B, Geller G, LeRoy L, Mathews DJH (2005). Opinions about new reproductive genetic technologies: hopes and fears for our genetic future. Fertil Steril.

[169] Bock von Wülfingen B (2016). Contested change: how Germany came to allow PGD. Reprod Biomed Soc Online.

[170] PGD conditions | Human Fertilisation and Embryology Authority. Available from: https://www.hfea.gov.uk/pgd-conditions/. Cited 16 Apr 2020.

[171] Kim JY, Lee HS, Kang IS (2015). Preimplantation genetic diagnosis. J Korean Med Assoc.

[172] Ginoza Margaret E.C., Isasi Rosario (2019). Regulating Preimplantation Genetic Testing across the World: A Comparison of International Policy and Ethical Perspectives. Cold Spring Harbor Perspectives in Medicine.

[173] Bayefsky MJ (2016). Comparative preimplantation genetic diagnosis policy in Europe and the USA and its implications for reproductive tourism. Reprod Biomed Soc Online.

[174] Benn P, Chapman AR (2016). Ethical and practical challenges in providing noninvasive prenatal testing for chromosome abnormalities: an update. Curr Opin Obstet Gynecol.

[175] Clarke AJ, Wallgren-Pettersson C (2019). Ethics in genetic counselling. J Community Genet.

[176] James CA, Hadley DW, Holtzman NA, Winkelstein JA (2006). How does the mode of inheritance of a genetic condition influence families? A study of guilt, blame, stigma, and understanding of inheritance and reproductive risks in families with X-linked and autosomal recessive diseases. Genet Med.

[177] Nazareth SB, Lazarin GA, Goldberg JD (2015). Changing trends in carrier screening for genetic disease in the United States. Prenat Diagn.

[178] Dollar EP. No easy choice : a story of disability, parenthood, and faith in an age of advanced reproduction. Louisville: Westminster John Knox Press; 2012.

[179] Wilkinson D, de Crespigny L, Xafis V (2014). Ethical language and decision-making for prenatally diagnosed lethal malformations. Semin Fetal Neonatal Med.

[180] Vanstone M, King C, de Vrijer B, Nisker J (2014). Non-invasive prenatal testing: ethics and policy considerations. J Obstet Gynaecol Can.

[181] Non-invasive prenatal testing - The Nuffield Council on Bioethics. Available from: https://nuffieldbioethics.org/publications/non-invasive-prenatal-testing. Cited 16 Jan 2020.

