# Supplementary material for: Reproductive options for families at risk of Osteogenesis Imperfecta: a review
Source: Orphanet J Rare Dis. 2020 May 27;15:128. doi: 10.1186/s13023-020-01404-w (PMC7251694; doi:10.1186/s13023-020-01404-w)
Supplement: Supplementary file 1 — Additional file 1: Table S1. Availability and legal regulations of reproductive techniques for families at risk of Osteogenesis Imperfecta across countries. § - Criteria of a disorder allowed for PGT-M is suggested by guidelines of professional organizations [172]. [file 13023_2020_1404_MOESM1_ESM.docx]

**Supplemental Table S1.** Availability and legal regulations of reproductive techniques for families at risk of Osteogenesis Imperfecta across countries.

| **#** | **Country** | **PGT-M** | **IVF with donor sperm** | **IVF with donor egg cell** | **IVF with donor embryo** |
| --- | --- | --- | --- | --- | --- |
| 1. | Australia | Approved, based on welfare of embryo, significance of the risk, severity and seriousness of the disorder. § | Anonymous | Anonymous | Allowed |
| 2. | Austria | Approved, based on severity and seriousness of the disorder. | Non-anonymous | Non-anonymous | Prohibited |
| 4. | Brazil | App roved. Genetic defect, no severity criteria. § | Non-anonymous | Non-anonymous | Allowed |
| 5. | Canada | Approved, based on severity and seriousness of the disorder. § | Anonymous | Anonymous | Allowed |
| 6. | China | Approved. | Anonymous | Anonymous | Allowed |
| 7. | Estonia | Approved, based on severity and seriousness of the disorder. | Anonymous | Anonymous | Allowed |
| 8. | Finland | Approved, based on severity and seriousness of the disorder. | Non-anonymous | Non-anonymous | Allowed |
| 9. | France | Approved, based on treatability, severity and seriousness of the disorder. | Anonymous | Anonymous | Allowed |
| 10. | Germany | Approved, based on treatability, severity and seriousness of the disorder. | Non-anonymous | Prohibited | Prohibited |
| 11. | India | Approved. Genetic defect, no severity criteria. | Anonymous | Anonymous | Allowed |
| 12. | Israel | Approved. § | Anonymous | Anonymous | Allowed |
| 13. | Italy | Approved, based on welfare of embryo. | Anonymous | Anonymous | Prohibited |
| 14. | Japan | Approved, based on severity and seriousness of the disorder. § | Anonymous | Anonymous | Allowed |
| 15. | The Netherlands | Approved, based on treatability, severity and seriousness of the disorder. | Non-anonymous | Non-anonymous | Allowed |
| 16. | South Korea | Approved for a set of conditions, including Osteogenesis Imperfecta. | Anonymity optional | Anonymity optional | NA |
| 17. | Norway | Approved, based on significance of the risk, treatability, severity and seriousness of the disorder. | Non-anonymous | Prohibited | Prohibited |
| 18. | Poland | Approved, based on significance of the risk, treatability, severity and seriousness of the disorder. | Anonymous | Anonymous | Allowed |
| 19. | Russia | Approved. Hereditary disorder. | Anonymous | Anonymous | Allowed |
| 20. | Spain | Approved for serious hereditary disorders without postnatal treatment. | Anonymous | Anonymous | Allowed |
| 21. | Switzerland | Approved, based on welfare of embryo, significance of the risk, treatability, severity and seriousness of the disorder. | Anonymous | Prohibited | Prohibited |
| 22. | Ukraine | Approved. Hereditary disorders. | Anonymous | Anonymous | Allowed |
| 23. | United Kingdom | Approved for a set of conditions, including Osteogenesis Imperfecta types I-XIII. | Non-anonymous | Non-anonymous | Allowed |
| 24. | US | Approved. Genetic defect, no severity criteria. § | Non-anonymous | Non-anonymous | Allowed |

§ - Criteria of a disorder allowed for PGT-M is suggested by guidelines of professional organizations [174].
